# Association between traffic-related air pollution and brain morphology, assessing the olfactory pathway as a mediator

**DOI:** 10.1093/braincomms/fcag221

**Published:** 2026-06-11

**Authors:** Margarethe Woeckel, Susanne Rospleszcz, Gizem Abaci, Maximilian Schwarz, Susanne Breitner-Busch, Fabian Bamberg, Michael Ingrisch, Christopher L Schlett, Kathrin Wolf, Alexandra Schneider, Sophia Stoecklein, Annette Peters

**Affiliations:** Institute of Epidemiology, German Research Center for Environmental Health, Helmholtz Zentrum München GmbH, 85764 Neuherberg, Germany; Department of Psychiatry and Psychotherapy, LMU University Hospital, LMU Munich, 80336 Munich, Germany; German Center for Mental Health (DZPG), Partner Site Munich/Augsburg, 80336 Munich, Germany; Institute of Epidemiology, German Research Center for Environmental Health, Helmholtz Zentrum München GmbH, 85764 Neuherberg, Germany; Department of Diagnostic and Interventional Radiology, Medical Center, Faculty of Medicine, University of Freiburg, 79106 Freiburg, Germany; Department of Radiology, LMU University Hospital, LMU Munich, 81377 Munich, Germany; Institute of Epidemiology, German Research Center for Environmental Health, Helmholtz Zentrum München GmbH, 85764 Neuherberg, Germany; Institute of Epidemiology, German Research Center for Environmental Health, Helmholtz Zentrum München GmbH, 85764 Neuherberg, Germany; Chair of Epidemiology, Institute for Medical Information Processing, Biometry and Epidemiology, Medical Faculty, LMU Munich, 81377 Munich, Germany; Department of Diagnostic and Interventional Radiology, Medical Center, Faculty of Medicine, University of Freiburg, 79106 Freiburg, Germany; Department of Radiology, LMU University Hospital, LMU Munich, 81377 Munich, Germany; Department of Diagnostic and Interventional Radiology, Medical Center, Faculty of Medicine, University of Freiburg, 79106 Freiburg, Germany; Institute of Epidemiology, German Research Center for Environmental Health, Helmholtz Zentrum München GmbH, 85764 Neuherberg, Germany; Institute of Epidemiology, German Research Center for Environmental Health, Helmholtz Zentrum München GmbH, 85764 Neuherberg, Germany; Department of Radiology, LMU University Hospital, LMU Munich, 81377 Munich, Germany; Institute of Epidemiology, German Research Center for Environmental Health, Helmholtz Zentrum München GmbH, 85764 Neuherberg, Germany; German Center for Mental Health (DZPG), Partner Site Munich/Augsburg, 80336 Munich, Germany; Chair of Epidemiology, Institute for Medical Information Processing, Biometry and Epidemiology, Medical Faculty, LMU Munich, 81377 Munich, Germany

**Keywords:** traffic-related air pollution, cranial MRI, white matter lesions, olfactory bulb, cross-sectional study

## Abstract

Traffic-related air pollution (TRAP) is a major environmental risk factor with growing evidence linking long-term exposure to adverse brain morphology outcomes. A direct pathway for pollutant translocation to the brain via the olfactory bulb has been proposed, but in vivo its contribution to TRAP-related changes in brain morphology remains inconclusive. We investigated these questions using cranial magnetic resonance imaging (MRI) data. Based on cranial MRIs from the population-based ‘Cooperative Health Research in the Region of Augsburg’ cohort, we analyzed global and region-specific white matter lesions, cerebral microbleeds, brain volumes, and olfactory bulb signal intensity in 400 participants. Land-use regression models estimated residential long-term exposure to air pollutants, including particle number concentration, particulate matter with different diameters, coarse particulate matter with a diameter between 10 μm and 2.5 μm, absorbance of particulate matter with an aerodynamic diameter ≤2.5 μm, and nitrogen oxides. We used covariate-adjusted regression models to explore the association between TRAP and white matter lesions, brain volumes, or cerebral microbleeds, and investigated whether the olfactory bulb mediates this association. Participants’ mean age was 56 ± 9 years, and 42% were female. We found that TRAP was associated with increased odds of prevalent white matter lesions. For global white matter lesions, an interquartile range increase in absorbance of particulate matter was associated with an odds ratio of 1.48 [95% CI: 1.02; 2.14]. Comparable results were found for non-frontal white matter lesions, where nitrogen dioxide, absorbance of particulate matter, and coarse particulate matter were linked to increased odds of white matter lesions. In the mediation analysis we did not find evidence that the association was mediated by the signal intensity of the olfactory bulb. Stratified analyses revealed that women were more susceptible for the detrimental effects of TRAP. Our findings suggest that TRAP exposure was associated with an increased odds for white matter lesions, while the olfactory bulb did not appear to mediate this relationship.

## Teaser

Woeckel *et al.* report that long-term exposure to traffic-related air pollution was associated with higher odds of white matter lesions on MRI in a population-based cohort, while no associations were observed for brain volumes. The olfactory bulb did not mediate this relationship, suggesting systemic mechanisms may underlie pollution-related brain alterations.

## Graphical Abstract

**Graphical Abstract:**
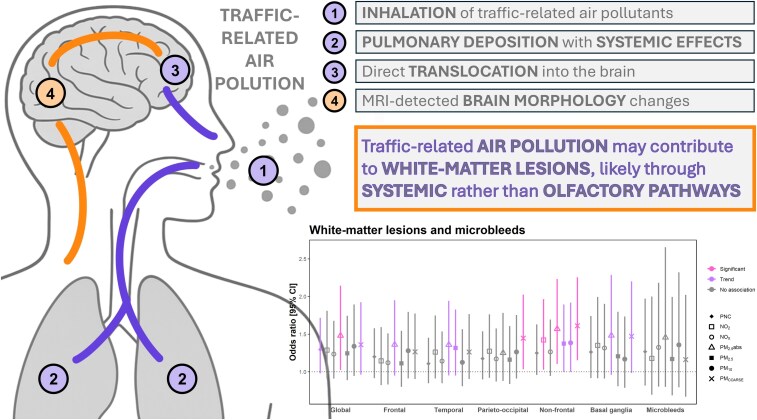

## Introduction

Air pollution is a significant global health concern, particularly in urban environments, where traffic-related air pollution (TRAP) accounts for a substantial share of overall ambient air pollution.^1,2^ TRAP, which encompasses both direct vehicle emissions and indirect sources such as tire and brake wear, consists of a complex mixture of air pollutants.^3^ Long-term exposure to TRAP, which includes ultrafine particles (UFP), black carbon (Bc), nitrogen dioxide (NO_2_), nitrogen oxides (NO_x_), and particulate matter (PM), has been associated with adverse effects on multiple organ systems, including the cardiovascular and respiratory systems.^4^ In recent years, increasing evidence suggests that TRAP may also play a significant role in neurological and mental health, contributing to both neurodevelopmental disorders and neurodegenerative diseases.^5-8^ However, the specific impact of TRAP on brain structure, particularly as measured by cranial magnetic resonance imaging (cMRI), remains incompletely understood,^9,10^ as most studies using cMRI data do not explicitly refer to traffic-related emissions.^11-14^

Epidemiological and experimental research suggests that TRAP might be associated with structural changes in brain morphology, including reductions in total cerebral brain volume, hippocampal atrophy, and increased burden of white matter lesions (WML).^9,10,15^ Additionally, animal models demonstrate that TRAP exposure leads to fibre-specific white matter degradation, potentially accelerating neurodegeneration.^16^

Scientific evidence suggests that air pollutants enter the brain and affect its structure and function. Experimental studies have shown that air pollutants can deposit on the olfactory epithelium, translocate along the olfactory nerve, and induce neuropathological changes,^17^ leading to their accumulation in the olfactory bulb and in deeper brain structures.^18-21^ Beyond direct translocation, air pollution can also induce neurotoxicity via systemic inflammation and oxidative stress.^22^ Inhaled pollutants trigger peripheral circulating pro-inflammatory cytokines that compromise the blood-brain-barrier and promote neuroinflammatory cascades.^22,23^

However, research on the effects of TRAP on brain morphology remains limited, as most imaging studies do not specifically consider traffic-related emissions as the source of pollution. Among existing studies, the focus is primarily on particulate matter with an aerodynamic diameter ≤2.5 μm (PM_2.5_), NO_2_, and NO_x_, while other critical traffic-related pollutants, such as UFP and Bc, remain unexplored. Additionally, the direct translocation of pollutants via the olfactory bulb has not been systematically examined in vivo. This study investigated in an explorative fashion the associations between TRAP (NO_2_, NO_X_, particle number concentration (PNC) as a proxy for UFP, PM with an aerodynamic parameter ≤10 μm (PM_10_), PM with an aerodynamic parameter ≤2.5 μm (PM_2.5_), particles with an aerodynamic parameter between 2.5 and 10 μm (PM_coarse_), and PM_2.5_ absorbance (PM_2.5_abs) as a proxy for Bc) and brain morphology, using cMRI. Furthermore, we used mediation analysis to assess whether pollutants reach the brain through the olfactory pathway.

## Materials and methods

### Study population

Within the KORA S4 cohort (Cooperative Health Research in the Region of Augsburg), the KORA-MRI study was designed as a cross-sectional population-based imaging study embedded within the second follow-up (FF4). The study region encompasses two neighbouring districts (Aichach-Friedberg district and Augsburg district) as well as the city of Augsburg, located in southern Germany. Detailed descriptions of the KORA cohort's design and recruitment have been published previously.^24^

The second follow-up included 2279 participants and was conducted from June 2013 to September 2014, of whom 400 were enrolled in the KORA-MRI sub-study. Comprehensive details regarding the study design, as well as eligibility and exclusion criteria, have been reported elsewhere.^25^ Briefly, individuals were excluded if they had an impaired renal function, had a history of cardiovascular disease such as myocardial infarction, revascularization, or stroke, were over 72 years of age, or had any contraindications to MRI.

The KORA-MRI study received ethical approval from the institutional review board of the Ludwig-Maximilians-University Munich (LMU Munich), while the KORA FF4 study was approved by the ethics committee of the Bavarian Chamber of Physicians in Munich. Written informed consent was received from all participants.

### Covariate assessment

Anthropometric measurements were collected at the KORA study centre. Standardized questionnaires and interviews were used to gather data on medication use, health status, physical activity, social status, alcohol consumption, and smoking habits.^25^ An oral glucose tolerance test was performed in participants without manifest diabetes, after which they were classified into normoglycemia, prediabetes, or diabetes groups based on WHO criteria.^26^

### Outcome assessment

Within 3 months after the visit to the study centre, all participants of the MRI sub-study underwent a whole-body MRI examination. Imaging was performed at a single study site using a 3 Tesla MAGNETOM Skyra system (Siemens Healthineers, Erlangen, Germany) with an 18-channel body coil in combination with the table-mounted spine matrix coil. A comprehensive standardized imaging protocol was applied identically to all participants. Detailed acquisition parameters and sequence specifications have been described previously.^25,27^

The following cMRI parameters were used for the current analysis:

- White matter lesions (WML) [yes/no], graded on T2 weighted fluid-attenuated inversion recovery (FLAIR) 3D images [SPACE, repetition time (TR): 5000 ms, slice thickness (ST): 0.9 mm, 0.5 mm × 0.5 mm in-plane spatial resolution, echo time (TE): 389 ms, flip angle: 120°, inversion time (TI): 1800 ms]:
  - Region-specific WML (frontal, temporal, perieto-occipital, infratentorial, non-frontal, basal ganglia).
  - Global WML.
  - Age-related WML; defined according to the Age-Related White Matter Changes (ARWMC) scale as WML are typically observed in older individuals and associated with aging and vascular risk factors.^28^
- Cerebral microbleeds [yes/no], derived from Susceptibility-Weighted Imaging (SWI).
- Cranial volumes [mm^3^], derived from T2 weighted FLAIR 3D images:
  - White matter (WM) volume.
  - Grey matter (GM) volume.
  - Cerebro-spinal fluid (CSF) volume.
  - Hippocampus and amygdala volume.
  - Total intracranial volume (ICV) (sum of WM volume, GM volume, and CSF volume).

A detailed description of how these measurements were obtained is presented in Supplementary Materials 1.

Image analyses were performed by trained readers, non-board-certified readers were closely supervised by board-certified radiologists. Readers were blinded to clinical and exposure information.

All images were reviewed by board-certified radiologists for clinically relevant incidental findings according to prospectively defined recommendations. Participants with clinically relevant findings were informed by standardized written notification including recommendations for further diagnostic work-up, as described previously.^29^

**Figure 1:**
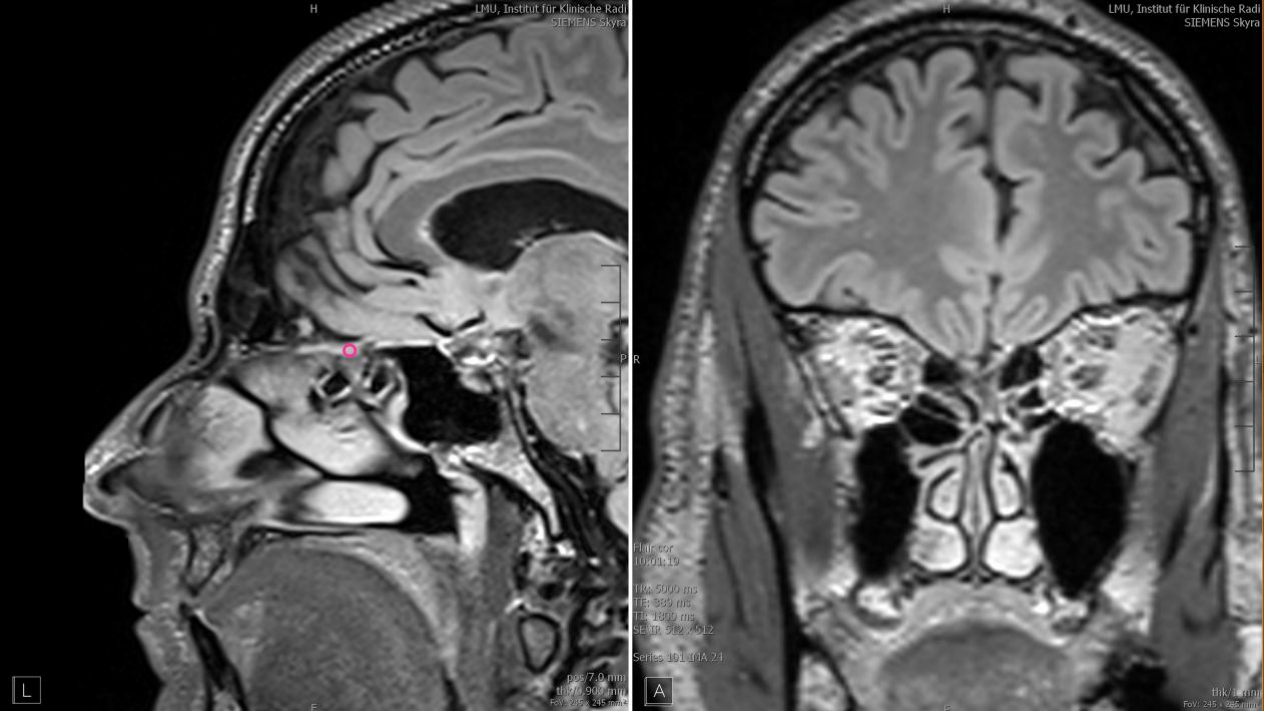
**Measurements of the signal intensity of the olfactory bulb**. In the sagittal plane on the left, the posterior third has been predefined as ROI (marked pink). The mean, minimum, and maximum signal intensity in arbitrary units were 178.14, 154, and 189 respectively. The coronal plane on the right serves as a reference.

For the current investigation, signal intensity in the olfactory bulb was bilaterally quantified using an approved and certified diagnostic monitor. Measurements with T2-weighted 3D FLAIR images [SPACE, repetition time (TR): 5000 ms, slice thickness (ST): 0.9 mm, 0.5 mm × 0.5 mm in-plane spatial resolution, echo time (TE): 389 ms, flip angle: 120°, inversion time (TI): 1800 ms] were performed in the sagittal plane, with the coronal plane serving as the reference. The posterior third of the olfactory bulb was predefined as the region of interest (ROI) (Fig. 1). Within the ROI, the minimum, mean, and maximum signal intensities in arbitrary units were extracted.

Inter-rater variability was assessed for 10% of the observations by trained physicians (MW, GA), under supervision of a board-certified radiologist (SS). Measurements were performed independently and readers were blinded to each other’s results. Mean signal intensity of the olfactory bulbs on both sides demonstrated low inter-rater variability (5.5% and 5.8%, respectively), and the variability for the minimum and maximum signal intensities ranged from 10% (maximum signal intensity) up to 35% (minimum signal intensity) (Supplementary Materials 2). In addition, intra-rater variability was assessed for 10% of the observations (Supplementary Materials 2). Intraclass correlation coefficients indicated moderate reproducibility overall, with higher agreement observed for mean and maximum signal intensity measures compared with minimum values (Supplementary Materials 2).

For signal normalization, cerebrospinal fluid (CSF) signal intensity was measured in the anterior horn of the right lateral ventricle. If measurement in the right ventricle was not feasible, the anterior horn of the left lateral ventricle was used as an alternative site. All signal intensities of the olfactory bulb were normalized for the CSF signal intensity. Mean, maximum, and minimum signal intensity of the olfactory bulb were used as outcomes in the current analysis.

### Exposure assessment

Long-term air pollution exposure was assessed for all KORA FF4 participants using land-use regression (LUR) models to estimate annual mean concentrations of air pollutants between March 2014 and April 2015.^30^ Residential exposure levels were assigned to participants based on their home addresses. The LUR models were developed using air pollution measurements from 20 monitoring stations located in urban and rural areas within the study region, sampled for three 14-day periods across different seasons (cold, warm, and intermediate).

Particle number concentrations (PNC) were measured using a NanoScan SMPS Nanoparticle Sizer (model 3910, TSI, Shoreview, MN, USA) and GRIMM ultrafine particle counters (model EDM 465 UFPC, GRIMM Aerosol, Ainring, Germany). Nitrogen oxides (NO_x_ and NO_2_) were quantified using Ogawa passive samplers (Ogawa & Co., USA Inc.). PM_10_ and PM_2.5_ levels were determined with Harvard Impactors. PM_coarse_ was calculated as the difference between PM_10_ and PM_2.5_. PM_2.5_absorbance (PM_2.5_abs) was derived from reflectance measurements of PM filters. It served as a proxy for Bc, which is predominantly traffic-related in this study area due to the high proportion of diesel vehicles in Germany.^31-33^

The final LUR models performed well with adjusted R^2^ (leave-one-out cross-validation adjusted R^2^) ranging between 0.68 (0.55) for PM_coarse_ and 0.94 (0.89) for NO_2_ and included the following predictor variables:

- PNC: Total traffic load of all major roads within 50 m; Industrial, commercial and transport units within 300 m; Forest and seminatural areas within 100 m; Urban green, forest and seminatural areas within 500 m; Building density within 25 m.
- PM_10_: Road length of all major roads within 100 m; Road length of all roads within 1000 m; Industrial, commercial and transport units within 300 m; Urban green within 500 m; Building density within 25 m; Total traffic load of all major roads within 25 m.
- PM_2.5_: Road length of all roads within 50 m; Industrial, commercial and transport units within 300 m; Forest and seminatural areas within 1000 m; Artificial surfaces within 25 m; Industrial, commercial and transport units within 300 m.
- PM_coarse_: Artificial surfaces within 1000 m; Product of traffic intensity on nearest road and inverse distance to the nearest road and distance squared; Industrial, commercial and transport units within 300 m.
- NO_2_: Industrial, commercial and transport units within 5000 m; Road length of all major roads within 100 m; Industrial, commercial and transport units within 300 m; Forest and seminatural areas within 100 m; Industrial, commercial and transport units within 1000 m.
- NO_X_: Total traffic load of all major roads within 50 m; Urban green, forest and seminatural areas within 1000 m; Industrial, commercial and transport units within 300 m; Forest and seminatural areas within 100 m.
- PM_2.5_abs: Total traffic load within 100 m; Building density within 25 m; Industrial, commercial and transport units within 1000 m; Water bodies within 5000 m.

Detailed information on model development, validation, quality, and measurement techniques is available in a prior publication.^30^

### Statistical analysis

Distributions of continuous variables were first inspected visually for normality. For variables exhibiting a non-normal distribution, the median and interquartile range (IQR) were reported, while variables following a normal distribution were expressed as mean and standard deviation. Group comparisons by sex for both participant characteristics and air pollution metrics were performed using Wilcoxon rank-sum tests, Chi-square tests, or *t*-tests, as appropriate.

Associations for each exposure-outcome pair were evaluated using covariate-adjusted regression models. We used linear regression for continuous outcome variables, correcting GM, WM, and CSF volumes by dividing each by ICV, and applying a square-root transformation to age-related WML. For binary WML outcomes, we used logistic regression models.

Covariate selection for adjustment was based on prior evidence from current literature. The minimum model was adjusted for sex and age, while the main model was additionally adjusted for alcohol consumption (g/day), body-mass index (BMI; in kg/m^2^), physical activity (very active, moderate active, little active, non-active), smoking (regular, former, never), marital status, and years of education.

For all exposure-outcome pairs, we performed mediation analysis for potential mediation by the mean or maximum signal intensity of the olfactory bulb. The mediation analysis was conducted with the covariate adjustment used in the main model with the R package ‘mediate’.

We performed stratified analyses to evaluate potential effect modifications. Following covariate adjustment of the main model, variables used to define the strata were not included as covariates in the respective stratified models: (I) sex female versus male, (II) normoglycemia versus prediabetes versus diabetes, (III) age <65 years versus ≥ 65 years, (IV) BMI < 30 kg/m^2^ versus ≥30 kg/m^2^, (V) hypertension no versus yes, (VI) high-sensitive C—reactive protein (hs-CRP) <1 mg/L versus ≥1 mg/L, participants with hs-CRP >10 mg/L were excluded.

We performed several sensitivity analyses to evaluate the robustness of the results, following covariate adjustment of the main model: Additional adjustment for (I) the degree of urbanization; (II) hs-CRP; (III) ICV. (IV) Instead of stratification, we used an interaction term between the respective air pollutant and the effect modifier. (V) Exclusion of all participants who had moved during the year of the study or in the preceding year.

Results are presented as either the percentage change of the mean outcome for linear regression models, as regression coefficients for outcome age-related WML, or as odds ratios (OR), and 95%-confidence intervals per interquartile range (IQR) increase in the respective air pollutant. A complete case analysis was used based on the availability of outcome data since exposure data were available for every participant.

Statistical analyses were executed using R version 4.3.1 (The R Foundation for Statistical Computing, Vienna, Austria). Due to the exploratory character of our analysis, we denote associations between exposure and outcome with a *P*-value < 0.1 as a trend, and those with a *P*-value < 0.05 as significant associations.

## Results

### Study population and exposure

The study population had a mean age of 56 ± 9 years, with 42% being female (Table 1). 40% of the participants had diabetes or prediabetes, and 34% had hypertension.

**Table 1:** Description of participants’ characteristics

Description of participants’ characteristics All participants: *n* = 400			
	Mean (SD)	Household income per month	*N* (%)
**Age** [years]	56.3 (9.2)	<625€	14 (4%)
**Weight** [kg]	83.0 (16.6)	625€ to <1250€	106 (27%)
**Height** [cm]	171.6 (9.7)	1250€ to <1875€	192 (48%)
**Waist circumference** [cm]	98.6 (14.3)	1875€ to <2500€	11 (3%)
**BMI** [kg/m^3^]	28.1 (4.9)	≥2500€	59 (14%)
**WHR**	0.9 (0.1)	Missing	18 (4%)
**SBP** [mmHg]	120.6 (16.7)	**Marital status**	
**DBP** [mmHg]	75.3 (10.0)	Unmarried, living alone	39 (10%)
**PP** [mmHg]	71.3 (9.9)	Unmarried, living with the partner	15 (4%)
**Cholesterol** [mg/dL]	217.8 (36.3)	Married, living with the spouse	289 (72%)
**HDL** [mg/dL]	61.9 17.7)	Married, living apart	9 (2%)
**LDL** [mg/dL]	139.5 (32.9)	Divorced	31 (8%)
**TAG** [mg/dL]	131.5 (84.8)	Widowed	17 (4%)
**Neighbourhood SES**	22.4 (21.7)	**Years of education**	
	**Median (IQR)**	8	10 (3%)
**Alcohol consumption** [g/day]	8.5 (25.7)	10	137 (34%)
**hsCRP** [mg/L]	1.2 (1.9)	11	55 (14%)
**Sex**	***N* (%)**	12	38 (9%)
Female	169 (42%)	13	80 (20%)
Male	231 (58%)	15	5 (1%)
**Diabetes status**		17	77 (19%)
Diabetes	54 (14%)	**Smoking habits**	
Prediabetes	103 (26%)	Regular	80 (20%)
Normoglycemia	243 (60%)	Former	174 (44%)
**Hypertension**	136 (34%)	Never	146 (36%)
**Angina pectoris**	25 (6%)	**Physical activity**	
**Antihypertensive medication**	102 (26%)	Very active	115 (29%)
**Lipid lowering medication**	43 (11%)	Moderate active	123 (31%)
**Antidiabetic medication**	32 (8%)	Little active	57 (14%)
		Non-active	105 (26%)

BMI, body mass index; DBP, diastolic blood pressure; HDL, high density lipoprotein; hsCRP, high sensitive c-reactive protein; IQR, interquartile range; LDL, low density lipoprotein; PP, pulse pressure; SBP, systolic blood pressure; SD, standard deviation; SES, socio-economic status; TAG, triacylglycerides; WHR, waist-to-hip ratio.

Details on cMRI outcomes and available sample size per outcome are summarized in Table 2. 62% of the participants exhibited WML, and the frontal lobe was most likely to be affected (60%). As WML in the infratentorial region were only detected in 14 participants, we did not assess this region as a dedicated outcome in regression analyses.

**Table 2:** Description of exposure and MRI outcome parameter

Description of environmental exposure All participants: *n* = 400			
	Mean (IQR)		Mean (IQR)
**PM_10_** [µg/m^3^]	16.5 (2.1)	**NO_2_** [µg/m^3^]	13.6 (6.0)
**PM_2.5_** [µg/m^3^]	11.7 (1.4)	**NO_X_** [µg/m^3^]	21.1 (9.7)
**PM_coarse_** [µg/m^3^]	4.8 (1.5)	**PM_2.5_abs** [10^−5^ m^−1^]	1.2 (0.3)
**PNC** [n/cm^3^]	7076.8 (2241.8)		

Description of cranial MRI parameter			
WML and cerebral microbleeds Included participants: *n* = 379	*N* (%)	Cranial volumes Included participants: *n* = 352	Mean (SD)
Global WML	249 (62.3)	Brain volume^a^	0.829 (0.028)
Frontal WML	238 (59.5)	GM volume (all reliable)^a^	0.076 (0.005)
Temporal WML	144 (36.0)	WM volume^a^	0.411 (0.016)
Parieto-occipital WML	145 (36.2)	CSF volume^a^	0.171 (0.028)
Infratentorial WML	14 (3.5)	Hippocampus and Amygdala volume^a^	0.008 (0.001)
Non-frontal WML	187 (46.8)	**Olfactory bulb signal intensity** Included participants: *n* = 322	**Mean (SD)**
Basal ganglia WML	66 (16.5)	Olfactory bulb mean^b^	7.66 (3.06)
Cerebral microbleedings	34 (8.5)	Olfactory bulb minimum^b^	6.23 (2.59)
**WML age-related** Included participants: *n* = 388	**Median (IQR)**	Olfactory bulb maximum^b^	8.75 (3.49)
Age-related WML	2 (5)		

CSF, cerebro-spinal fluid; GM, grey-matter; NO_2_, nitrogen dioxide; NO_X_, nitrogen oxides; PM_10_, particulate matter with an aerodynamic diameter ≥10 μm; PM_2.5_, particulate matter with an aerodynamic diameter ≥2.5 μm; PM_2.5_abs, PM_2.5_ absorbance; PMcoarse, particles with an aerodynamic parameter 10–2.5 μm; PNC, particle number concentration; WM, white matter; WML, white matter lesions.

^a^Corrected for ICV.

^b^Corrected for cerebro-spinal fluid signal intensity.

Air pollution exposures and outcomes did not differ significantly between women and men (Supplementary Table 1). The average annual PM_2.5_ exposure was 11.7 µg/m^3^, with an interquartile range (IQR) of 1.4 µg/m^3^ (Table 2). This exposure level is below the EU annual threshold of 25 µg/m^3^. Similarly, average NO_2_ levels (13.6 µg/m^3^) were within the EU limit of 40 µg/m^3^. PM_10_ levels averaged 16.5 µg/m^3^, remaining under the EU limit of 40 µg/m^3^.^34^ Although the revised Ambient Air Quality Directive (AAQD) will lower the annual limits for PM_2.5_ and NO_2_ from 2030 onward, the observed exposure levels in this study were also below these new thresholds. Air pollutants were highly to moderately correlate with each other (Supplementary Table 2).

### TRAP and brain morphology

**Figure 2:**
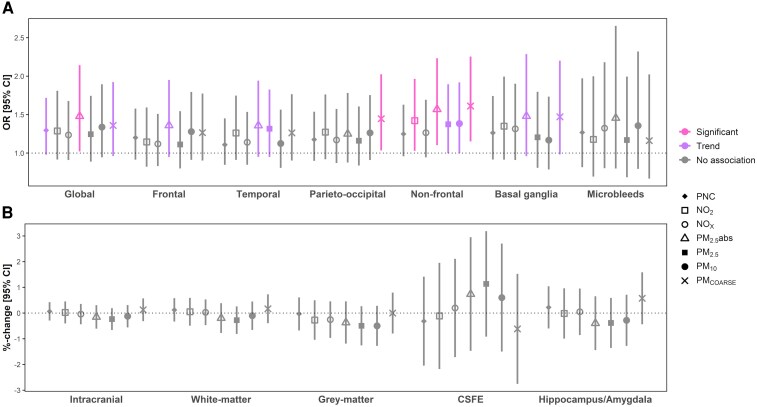
**Associations between TRAP and brain morphology**. The upper panel (**A**) presents odd ratios (OR) and 95%-confidence intervals (CI) for global and region-specific WML, and cerebral microbleeds per interquartile-range (IQR) increase in the respective air pollutant (*n* = 379). The lower panel (**B**) presents %-changes and 95%-CI for brain volumes per IQR increase in the respective air pollutant (*n* = 352). Strength of association derived from the regression analysis: significant: *P*-value < 0.05. Trend: 0.1 > *P*-value ≥ 0.05. No association: *P*-value ≥ 0.1. Models were adjusted for age, sex, BMI, alcohol consumption, smoking, physical activity, marital status and education years. White matter, grey matter, cerebro-spinal fluid (CSFE), and Hippocampus/Amygdala volumes were corrected for ICV.

We found that exposure to TRAP was associated with an increased odds for the presence of WML (Fig. 2, Supplementary Table 3). In global WML, an IQR increase in PM_2.5_abs was associated with an increased OR of 1.48 [95%-CI: 1.02; 2.14]. Comparable results were found for non-frontal WML, where NO_2_, PM_2.5_abs, and PM_coarse_ were linked to increased odds for WML [NO_2_: 1.42 (1.03; 1.96); PM_2.5_abs: 1.57 (1.10; 2.23); PM_coarse_: 1.61 (1.15; 2.25)]. For other air pollutants, such as PNC and PM_2.5_, we observed trends towards increased odds for the presence of WML (Fig. 2, Supplementary Table 3), while we did not find an association between TRAP and the presence of microbleeds. PM_2.5_abs and PM_coarse_ were associated with increased age-related WML (Table 3). For example, an IQR increase in PM_2.5_abs was associated with a 0.17 [0.01; 0.33] increase in square-root-transformed age-related WML. There were no significant associations between TRAP and any brain volumes (Fig. 2, Supplementary Table 3).

**Table 3:** Associations between TRAP and square-root age-related WML

Exposure	IQR	Coeff [95%-CI]	*P*-value
PNC	2241.76	0.09 [−0.03; 0.22]	*0*.*135*
NO_2_	6.03	0.10 [−0.05; 0.25]	*0*.*198*
NO_X_	9.67	0.07 [−0.07; 0.20]	*0*.*328*
PM_2.5_abs	0.28	0.17 [0.01; 0.33] *	*0*.*036*
PM_2.5_	1.41	0.05 [−0.10; 0.20]	*0*.*491*
PM_10_	2.06	0.11 [−0.04; 0.26]	*0*.*154*
PM_coarse_	1.47	0.15 [0.00; 0.30] *	*0*.*048*

Results are presented as regression coefficients (coeff) and 95% confidence intervals [95%-CI] per interquartile-range (IQR) increase in the respective air pollutant. Square-root regression models (SQRM) were adjusted for age, sex, BMI, alcohol consumption, smoking, physical activity, marital status, and education years. Significant results are indicated by *.

### Mediation by the olfactory bulb

In the mediation analysis, we did not find evidence that the mean or maximum signal intensity of the olfactory bulb acts as a mediator between TRAP and WML or brain volumes (Supplementary Figs 1–4). The total effect for the association between TRAP and global or region-specific WML and volumes (Supplementary Figs 1 and 2) was comparable to the associations found in the main analysis (Fig. 2, Supplementary Table 3). However, the average mediation effect, which represents the effect between exposure and outcome that is mediated by the signal intensity of the olfactory bulb, was close to 0 and not significant for any of the examined associations, the same applies to the proportion mediated (results not shown).

### Effect modification

**Figure 3:**
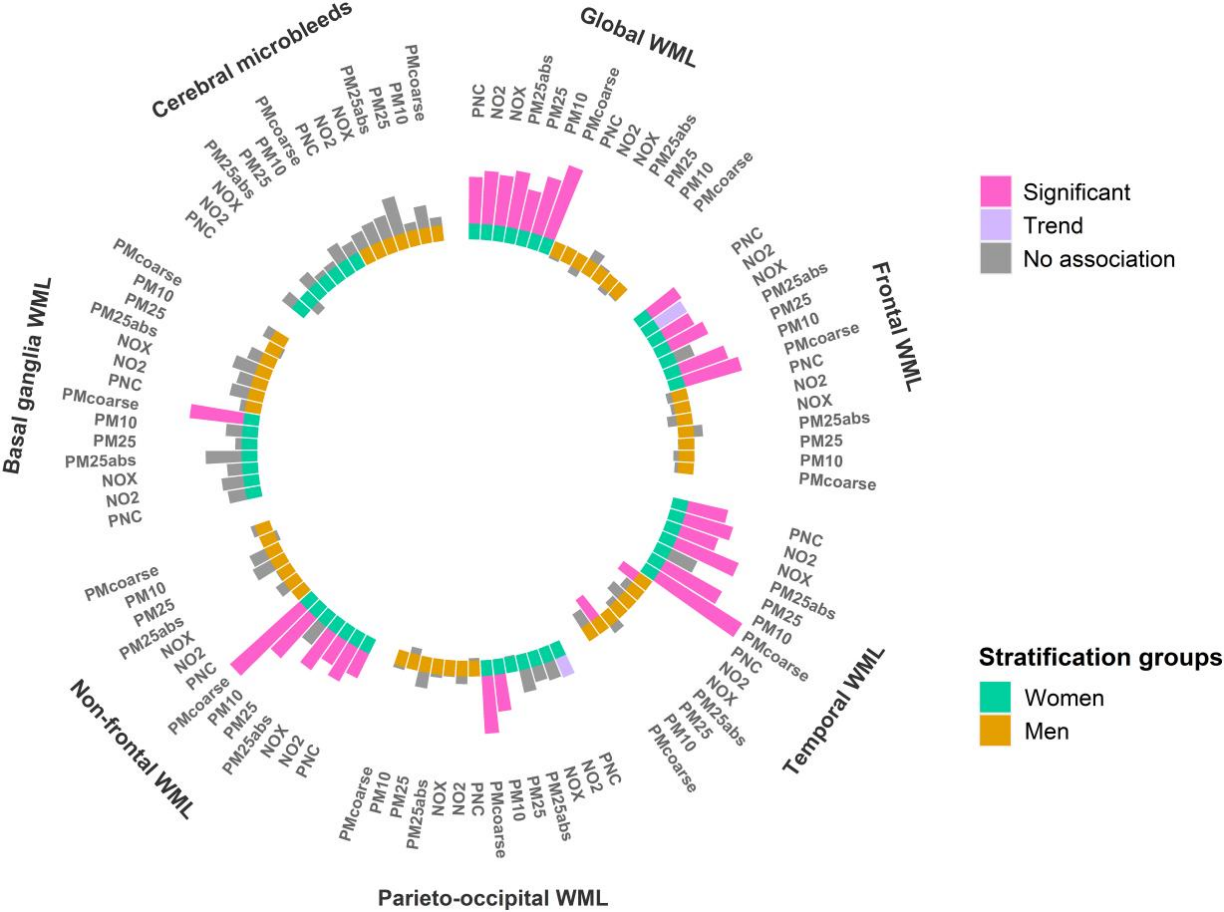
**Associations between TRAP and global and region-specific white matter lesions (WML), and cerebral microbleeds, stratified by sex**. The height of the bars indicates the odds ratio (OR) per IQR increase in the respective air pollutant. The direction of the bars indicates the direction of the association. Bars toward the centre indicate a negative association (OR < 1), bars toward outside a positive association (OR > 1). Strength of association derived from the regression analysis: significant: *P*-value < 0.05. Trend: 0.1 > *P*-value ≥ 0.05. No association: *P*-value ≥ 0.1. Models were adjusted for age, BMI, alcohol consumption, smoking, physical activity, marital status and education years. *n* = 379.

Stratification by sex significantly influenced the observed associations. We observed a significant association between TRAP exposure and the presence of WML in women compared with men. Women exhibited an increased odds of WML, both global and region-specific in the frontal, temporal, and parieto-occipital regions, for nearly all air pollutants (Fig. 3). In contrast, no significant associations were found in men. For brain volumes, we found similar results. While no associations were detected in the main analysis, stratification revealed that exposure to TRAP was associated with lower white matter volumes only in women (Supplementary Fig. 5). Additionally, we observed a trend toward decreased grey matter volume and total ICV.

Other stratification analyses yielded less consistent results (Supplementary Figs 6–8). Notably, individuals with diabetes had significantly decreased odds of WML in the temporal, parieto-occipital, and non-frontal regions, whereas participants with prediabetes exhibited higher odds (Supplementary Fig. 6). Stratification by BMI did not reveal significant differences in TRAP-associated WML odds between participants with BMI ≥ 30 kg/m^2^ and those with BMI < 30 kg/m^2^ (Supplementary Fig. 7).

### Sensitivity analysis

The results remained largely stable across most sensitivity analyses (Supplementary Tables 4–7). Adjusting the main models for hs-CRP did not significantly alter the observed associations. Similarly, additional adjustment for ICV did not impact the findings. The same applies to the adjustment for the degree of urbanization, or the exclusion of movers. Using an interaction term for effect modification instead of stratification also yielded consistent results, further supporting the robustness of the conducted analyses.

## Discussion

In our cross-sectional study, we examined in an explorative fashion the association between long-term exposure to TRAP and brain morphology, using data from a population-based MRI cohort study. We found that exposure to TRAP was significantly associated with higher odds of prevalent WML. In particular, PM_2.5_abs showed significant associations with global and region-specific WML. In contrast, no significant associations were observed for brain volumes. Mediation analysis provided no evidence that the signal intensity of the olfactory bulb acts as a mediator between TRAP exposure and brain morphology. In the stratified analyses, women had significantly higher odds for present WML and reduced brain volumes compared with men.

WML are widely recognized as imaging markers of cerebral small vessel disease. A substantial body of evidence demonstrates that WML are associated with an increased risk of recurrent stroke and major vascular events, as well as with long-term cognitive decline and incident dementia, particularly vascular dementia.^35-37^ In addition, WML have been linked to late-life and post-stroke depression, supporting the concept of a vascular contribution to neuropsychiatric outcomes.^38,39^ Thus, the observed association between TRAP and WML in our study may have important clinical implications, as WML represent a structural marker underlying stroke, dementia, and affective disorders. As TRAP is a potentially modifiable environmental exposure, our findings suggest that reducing long-term exposure may contribute to lowering the burden of brain injury. From a clinical and public health perspective, this highlights the importance of environmental risk factor control as part of broader strategies aimed at preventing stroke, cognitive decline, and neuropsychiatric disorders at the population level.

Previous studies have reported inconsistent evidence regarding the association between air pollution exposure and the risk of WML. While the Three-City Montpellier study reported positive associations PM_2.5_ and NO_2_ and region-specific WML,^14^ other cohort studies did not confirm such relationships.^10,11,15,40^ Similarly, analyses from the Framingham Offspring Study on residential proximity to major roadways did not identify a clear pattern of association with WML.^9^

Contrary to our hypothesis, we did not observe significant associations between TRAP exposure and brain volumes. Previous studies have reported inconsistent findings regarding air pollution and brain volumes.^11-13,15,41^ Thus, our results for volumetric measures were consistent with the heterogeneous evidence in the literature. However, we observed TRAP-related associations for markers of small vessel disease, such as WML, but not for brain volumes, suggesting that air pollution effects in this population may preferentially manifest as microvascular WM alterations rather than global volumetric changes.

Understanding the mechanisms through which air pollution affects brain health is crucial for assessing its long-term neurophysiological consequences. Existing literature highlights two major pathways through which air pollution can impact brain health: direct neuroinvasion via the olfactory system and indirect effects through systemic inflammation and oxidative stress.^22^ Animal studies have demonstrated that inhaled UFP, metal-laden nanoparticles, and Bc can translocate from the nasal cavity to the olfactory bulb and subsequently distribute even to deeper brain regions, inducing neuroinflammatory responses.^18,19^ Autopsy studies provided evidence that chronic air pollution exposure is associated with neuropathological changes in the olfactory bulb, including early neurodegenerative markers such as hyperphosphorylated tau and amyloid plaques.^21,42^ However, despite strong evidence supporting olfactory translocation, its contribution to broader neurotoxic effects within the brain remains debated, particularly in the context of widespread WM alterations.^17,18,22^

Systemic pathways appear to play a major role in air pollution-induced neurotoxicity. Exposure to particulate matter, diesel exhaust, and nanoparticles has been linked to systemic inflammation, oxidative stress, and cerebrovascular damage, all of which can contribute to neurodegeneration and structural brain changes.^43,44^ Even in cases where direct olfactory translocation is limited, air pollution exposure can disrupt cognitive function and brain integrity via inflammatory cascades originating in the lungs and systemic circulation.^23^ The ability of UFP to cross the blood-brain barrier further complicates this interplay, as circulating pollutants and inflammation-related mediators may exert neurotoxic effects independently of direct neuronal translocation.^17,22,44^

Our study did not find evidence for a significant mediation effect of olfactory bulb signal intensity in the association between TRAP exposure and brain morphology. This might be a hint that the increased risk of developing WML identified in our main analysis is more likely driven by systemic mechanisms rather than direct translocation of particles via the olfactory nerve. However, it is also possible that olfactory bulb signal intensity does not adequately capture the relevant neuroanatomical or pathological changes necessary to detect direct neuroinvasion. Alternative markers, such as volumetric measures of the olfactory bulb, microstructural integrity, or the presence of olfactory bulb lesions, may provide a more accurate assessment of its potential involvement in air pollution-related neurotoxicity.

Our findings complement recent large-scale neuroimaging analyses from the UK Biobank, which reported inverse associations between long-term exposure to particulate matter and nitrogen oxides and total grey and white matter volumes,^41^ well as positive associations with white matter hyperintensity burden.^45^ While we did not observe significant associations for brain volumes, we identified significant associations between TRAP and WML presence, suggesting that pollution-related effects in our cohort may be primarily reflected in microvascular white matter alterations rather than global atrophy measures. Ongoing and future analyses within other deeply phenotyped population-based cohorts, such as the German National Cohort (NAKO), will be important to replicate and extend these findings, particularly with respect to WML burden and brain volumes.

A major strength of this study is its integration within a well-characterized cohort, allowing for comprehensive model adjustments based on a wide range of covariates. This rigorous design enhances the reliability of our findings and provides valuable insights into the potential mechanisms. Furthermore, the use of cMRI ensures highly precise and reproducible assessments, as it is considered the gold standard for evaluating structural brain changes. Unlike previous research, which has mainly focused on selected air pollutants, we had a more comprehensive approach by examining the association between a broad spectrum of TRAP exposures and detailed brain morphology measures. By assessing global and region-specific WML, including age-related patterns, as well as volumetric brain measures, this study enables a more detailed exploration of potential exposure-response relationships.

Several limitations should be considered when interpreting the findings. The cross-sectional design limits causal inference, and the study’s small geographic scope with limited racial diversity may affect generalizability. Participation in the MRI sub-study was restricted to eligible and willing members of the underlying cohort and further reduced by MRI-specific exclusion criteria and image quality requirements, which may introduce selection bias. However, previous analyses using sampling weights indicated that the MRI sample was broadly comparable to the eligible source population and that substantial selection bias is unlikely to have materially affected the main findings.^46^ TRAP was estimated based on residential addresses, without accounting for time spent elsewhere, such as workplaces or during commutes. This may have led to misclassification, especially for rural residents commuting to urban areas. With respect to TRAP exposure, our LUR models did not include a dedicated source apportionment analysis. Furthermore, the small sample size constrained statistical power. Multiple comparisons were conducted without adjustment for multiple testing, increasing the chance of spurious findings. Underlining the explorative character of the study, results were interpreted as patterns of association across related pollutants and outcomes.

## Conclusion

In our population-based MRI study, we found that long-term exposure to TRAP was associated with higher odds of prevalent WML, while no significant associations were observed for brain volumes. Mediation analysis did not support a contribution of the olfactory bulb in this association in our study, suggesting that, based on our findings, systemic inflammatory mechanisms may be the more likely pathway linking TRAP to brain changes. Our findings underline the importance of reducing long-term exposure to TRAP as a potential preventive strategy for brain health. At the same time, larger sample sizes and longitudinal studies are needed to confirm these associations and to further disentangle the underlying biological pathways.

## Supplementary Material

fcag221_Supplementary_Data

## Data Availability

The data that support the findings of this study are available on request from the corresponding author. The data are not publicly available because they contain sensitive participant information. R codes are provided in Supplementary Materials 3.

## References

[1] EU . Emissions of pollutants from transport in EU-27. Accessed 03.03.2025. https://www.eea.europa.eu/en/analysis/indicators/emissions-of-air-pollutants-from?activeAccordion=

[2] Reche C, Tobias A, Viana M. Vehicular traffic in urban areas: Health burden and influence of sustainable urban planning and mobility. Atmosphere (Basel). 2022;13(4):598.

[3] Khreis H, Nieuwenhuijsen MJ, Zietsman J, Ramani T. Chapter 1—Traffic-related air pollution: Emissions, human exposures, and health: An introduction. In: Khreis H, Nieuwenhuijsen M, Zietsman J, Ramani T, eds. Traffic-related air pollution. Elsevier; 2020:1–21.

[4] Boogaard H, Patton AP, Atkinson RW, et al Long-term exposure to traffic-related air pollution and selected health outcomes: A systematic review and meta-analysis. Environ Int. 2022;164:107262. 35569389 10.1016/j.envint.2022.107262

[5] Woodward N, Finch CE, Morgan TE. Traffic-related air pollution and brain development. AIMS Environ Sci. 2015;2(2):353–373. 27099868 10.3934/environsci.2015.2.353 PMC4835031

[6] Ritz B, Lee PC, Hansen J, et al Traffic-related air pollution and Parkinson's disease in Denmark: A case-control study. Environ Health Perspect. 2016;124(3):351–356. 26151951 10.1289/ehp.1409313 PMC4786985

[7] Paul KC, Haan M, Yu Y, et al Traffic-related air pollution and incident dementia: Direct and indirect pathways through metabolic dysfunction. J Alzheimers Dis. 2020;76(4):1477–1491. 32651321 10.3233/JAD-200320 PMC7591265

[8] Pedersen CB, Raaschou-Nielsen O, Hertel O, Mortensen PB. Air pollution from traffic and schizophrenia risk. Schizophr Res. 2004;66(1):83–85. 14693358 10.1016/s0920-9964(03)00062-8

[9] Wilker EH, Preis SR, Beiser AS, et al Long-term exposure to fine particulate matter, residential proximity to major roads and measures of brain structure. Stroke. 2015;46(5):1161–1166. 25908455 10.1161/STROKEAHA.114.008348 PMC4414870

[10] Kulick ER, Wellenius GA, Kaufman JD, et al Long-term exposure to ambient air pollution and subclinical cerebrovascular disease in NOMAS (the Northern Manhattan Study). Stroke. 2017;48(7):1966–1968. 28455324 10.1161/STROKEAHA.117.016672 PMC5487287

[11] Power MC, Lamichhane AP, Liao D, et al The association of long-term exposure to particulate matter air pollution with brain MRI findings: The ARIC study. Environ Health Perspect. 2018;126(2):027009. 29467108 10.1289/EHP2152 PMC6066342

[12] Casanova R, Wang X, Reyes J, et al A voxel-based morphometry study reveals local brain structural alterations associated with ambient fine particles in older women. Front Hum Neurosci. 2016;10:495. 27790103 10.3389/fnhum.2016.00495 PMC5061768

[13] Cho J, Noh Y, Kim SY, et al Long-term ambient air pollution exposures and brain imaging markers in Korean adults: The environmental pollution-induced neurological EFfects (EPINEF) study. Environ Health Perspect. 2020;128(11):117006. 33215932 10.1289/EHP7133 PMC7678746

[14] Duchesne J, Carrière I, Artero S, et al Ambient air pollution exposure and cerebral white matter hyperintensities in older adults: A cross-sectional analysis in the three-city montpellier study. Environ Health Perspect. 2023;131(10):107013. 37878794 10.1289/EHP12231 PMC10599635

[15] Lynch KM, Bennett EE, Ying Q, et al Association of gaseous ambient air pollution and dementia-related neuroimaging markers in the ARIC cohort, comparing exposure estimation methods and confounding by study site. Environ Health Perspect. 2024;132(6):67010. 38922331 10.1289/EHP13906 PMC11218707

[16] Chen TC, Lo YC, Li SJ, et al Assessing traffic-related air pollution-induced fiber-specific white matter degradation associated with motor performance declines in aged rats. Ecotoxicol Environ Saf. 2023;263:115373. 37619400 10.1016/j.ecoenv.2023.115373

[17] Calderón-Garcidueñas L, Reynoso-Robles R, González-Maciel A. Combustion and friction-derived nanoparticles and industrial-sourced nanoparticles: The culprit of Alzheimer and Parkinson's diseases. Environ Res. 2019;176:108574. 31299618 10.1016/j.envres.2019.108574

[18] Oberdörster G, Sharp Z, Atudorei V, et al Translocation of inhaled ultrafine particles to the brain. Inhal Toxicol. 2004;16(6–7):437–445. 15204759 10.1080/08958370490439597

[19] Elder A, Gelein R, Silva V, et al Translocation of inhaled ultrafine manganese oxide particles to the central nervous system. Environ Health Perspect. 2006;114(8):1172–1178. 16882521 10.1289/ehp.9030 PMC1552007

[20] Ajmani GS, Suh HH, Pinto JM. Effects of ambient air pollution exposure on olfaction: A review. Environ Health Perspect. 2016;124(11):1683–1693. 27285588 10.1289/EHP136 PMC5089874

[21] Calderón-Garcidueñas L, Maronpot RR, Torres-Jardon R, et al DNA damage in nasal and brain tissues of canines exposed to air pollutants is associated with evidence of chronic brain inflammation and neurodegeneration. Toxicol Pathol. 2003;31(5):524–538. 14692621 10.1080/01926230390226645

[22] You R, Ho YS, Chang RC. The pathogenic effects of particulate matter on neurodegeneration: A review. J Biomed Sci. 2022;29(1):15. 35189880 10.1186/s12929-022-00799-x PMC8862284

[23] Faherty T, Raymond JE, McFiggans G, Pope FD. Acute particulate matter exposure diminishes executive cognitive functioning after four hours regardless of inhalation pathway. Nat Commun. 2025;16(1):1339. 39915448 10.1038/s41467-025-56508-3 PMC11803098

[24] Holle R, Happich M, Löwel H, Wichmann HE. KORA–a research platform for population based health research. Gesundheitswesen. 2005;67(Suppl 1):S19–S25. 16032513 10.1055/s-2005-858235

[25] Bamberg F, Hetterich H, Rospleszcz S, et al Subclinical disease burden as assessed by whole-body MRI in subjects with prediabetes, subjects with diabetes, and normal control subjects from the general population: The KORA-MRI study. Diabetes. 2017;66(1):158–169. 27999110 10.2337/db16-0630

[26] WHO . Definition and diagnosis of diabetes mellitus and intermediate hyperglycaemia: report of a World Health Organisation/IDF consultation. 2006. Accessed 16.06.2026. https://iris.who.int/server/api/core/bitstreams/ef6a81ae-5db3-4c5c-9136-c047bd8f8344/content

[27] Grosu S, Rospleszcz S, Hartmann F, et al Associated factors of white matter hyperintensity volume: A machine-learning approach. Sci Rep. 2021;11(1):2325. 33504924 10.1038/s41598-021-81883-4 PMC7840689

[28] Wahlund LO, Barkhof F, Fazekas F, et al A new rating scale for age-related white matter changes applicable to MRI and CT. Stroke. 2001;32(6):1318–1322. 11387493 10.1161/01.str.32.6.1318

[29] Schlett CL, Rospleszcz S, Korbmacher D, et al Incidental findings in whole-body MR imaging of a population-based cohort study: Frequency, management and psychosocial consequences. Eur J Radiol. 2021;134:109451. 33279799 10.1016/j.ejrad.2020.109451

[30] Wolf K, Cyrys J, Harciníková T, et al Land use regression modeling of ultrafine particles, ozone, nitrogen oxides and markers of particulate matter pollution in Augsburg, Germany. Sci Total Environ. 2017;579:1531–1540. 27916311 10.1016/j.scitotenv.2016.11.160

[31] Eeftens M, Beelen R, de Hoogh K, et al Development of land use regression models for PM(2.5), PM(2.5) absorbance, PM(10) and PM(coarse) in 20 European study areas; results of the ESCAPE project. Environ Sci Technol. 2012;46(20):11195–11205. 22963366 10.1021/es301948k

[32] Cyrys J, Heinrich J, Hoek G, et al Comparison between different traffic-related particle indicators: Elemental carbon (EC), PM2.5 mass, and absorbance. J Expo Anal Environ Epidemiol. 2003;13(2):134–143. 12679793 10.1038/sj.jea.7500262

[33] Kraftfahrbundesamt . Jahresbilanz 2014. Accessed 28.07.2024. https://www.kba.de/DE/Statistik/Fahrzeuge/Bestand/Jahrebilanz_Bestand/fz_b_jahresbilanz_node.html?yearFilter=2014

[34] EU . Directive 2008/50/EC of the European parliament and of the council of 21 may 2008 on ambient air quality and cleaner air for Europe. Official Journal of the European Union. 2008;152:1–44.

[35] Park JH, Heo SH, Lee MH, Kwon HS, Kwon SU, Lee JS. White matter hyperintensities and recurrent stroke risk in patients with stroke with small-vessel disease. Eur J Neurol. 2019;26(6):911–918. 30637882 10.1111/ene.13908

[36] Hu HY, Ou YN, Shen XN, et al White matter hyperintensities and risks of cognitive impairment and dementia: A systematic review and meta-analysis of 36 prospective studies. Neurosci Biobehav Rev. 2021;120:16–27. 33188821 10.1016/j.neubiorev.2020.11.007

[37] Luo W, Dai Z, Wu W, Li H, Zhang Y. White matter hyperintensities and the risk of vascular dementia: A systematic review and meta-analysis. PeerJ. 2025;13:e19460. 40538738 10.7717/peerj.19460 PMC12178243

[38] Herrmann LL, Le Masurier M, Ebmeier KP. White matter hyperintensities in late life depression: A systematic review. J Neurol Neurosurg Psychiatry. 2008;79(6):619–624. 17717021 10.1136/jnnp.2007.124651

[39] Zhang F, Ping Y, Jin X, Hou X, Song J. White matter hyperintensities and post-stroke depression: A systematic review and meta-analysis. J Affect Disord. 2023;320:370–380. 36209775 10.1016/j.jad.2022.09.166

[40] Power MC, Lynch KM, Bennett EE, et al A comparison of PM(2.5) exposure estimates from different estimation methods and their associations with cognitive testing and brain MRI outcomes. Environ Res. 2024;256:119178. 38768885 10.1016/j.envres.2024.119178 PMC11186721

[41] Erickson LD, Gale SD, Anderson JE, Brown BL, Hedges DW. Association between exposure to air pollution and total gray matter and total white matter volumes in adults: A cross-sectional study. Brain Sci. 2020;10(3):164. 32182984 10.3390/brainsci10030164 PMC7139378

[42] Calderón-Garcidueñas L, González-Maciel A, Reynoso-Robles R, et al Alzheimer's disease and alpha-synuclein pathology in the olfactory bulbs of infants, children, teens and adults ≤ 40 years in metropolitan Mexico city. APOE4 carriers at higher risk of suicide accelerate their olfactory bulb pathology. Environ Res. 2018;166:348–362. 29935448 10.1016/j.envres.2018.06.027

[43] Levesque S, Surace MJ, McDonald J, Block ML. Air pollution & the brain: Subchronic diesel exhaust exposure causes neuroinflammation and elevates early markers of neurodegenerative disease. J Neuroinflammation. 2011;8:105. 21864400 10.1186/1742-2094-8-105 PMC3184279

[44] Wang Y, Xiong L, Tang M. Toxicity of inhaled particulate matter on the central nervous system: Neuroinflammation, neuropsychological effects and neurodegenerative disease. J Appl Toxicol. 2017;37(6):644–667. 28299803 10.1002/jat.3451

[45] Song T, Qiu X, Wu J, et al Associations of ambient air pollution exposure and sleep pattern with brain structures: A prospective study in the UK biobank. Ecotoxicol Environ Saf. 2025;302:118517. 40555092 10.1016/j.ecoenv.2025.118517

[46] Rospleszcz S, Schafnitzel A, Koenig W, et al Association of glycemic status and segmental left ventricular wall thickness in subjects without prior cardiovascular disease: A cross-sectional study. BMC Cardiovasc Disord. 2018;18(1):162. 30092757 10.1186/s12872-018-0900-7 PMC6085649

