# Supplementary material for: Association between traffic-related air pollution and brain morphology, assessing the olfactory pathway as a mediator
Source: Brain Commun. 2026 Jun 11;8(3):fcag221. doi: 10.1093/braincomms/fcag221 (PMC13281947; doi:10.1093/braincomms/fcag221)
Supplement: fcag221_Supplementary_Data [file fcag221_supplementary_data.pdf]

Association between traffic-related air pollution and brain morphology, assessing the olfactory pathway as a mediator.

Woeckel, Margarethe; Rospleszcz, Susanne; Abaci, Gizem; Schwarz, Maximilian; Breitner-Busch, Susanne; Bamberg, Fabian; Ingrisch, Michael; Schlett, Christopher L; Wolf, Kathrin; Schneider, Alexandra; Stoecklein, Sophia; Peters, Annette.

SUPPLEMENTARY MATERIAL

Supplementary Material 1 – MRI outcome variables measurement and definition

Brain volumes: Warp-based automated brain volumetry was performed using FLAIR MRI data, as T1-weighted images were unavailable in the KORA-MRI study due to time constraints. The feasibility of FLAIR-based volumetry was validated in a study by (Beller et al. 2019), which compared FLAIR- and T1-based measurements across 116 atlas regions in 30 healthy individuals. Image processing followed the validated pipeline: Preprocessing was done using FSL 5.0.9 (http://www.fmrib.ox.ac.uk/fsl/index.html) and AFNI (Analyses of Functional Images http://afni.nimh.nih.gov/afni). FLAIR images were reoriented to MNI152 space (FSL), skull-stripped (BET), and segmented into gray matter (GM), white matter (WM), and cerebrospinal fluid (CSF) using FAST. Images were then registered to the AAL atlas (FLIRT, FNIRT), and volumes for total grey-matter, white-matter, cerebro-spinal fluid, and 116 atlas regions were extracted.[1, 2]

Grey-matter volumes – all reliable measures:
Grey Matter Volume of AAL region Cingulum_Ant_R, residuals (in mm^3)
Grey Matter Volume of AAL region Cingulum_Mid_L, residuals (in mm^3)
Grey Matter Volume of AAL region Cingulum_Mid_R, residuals (in mm^3)
Grey Matter Volume of AAL region Cingulum_Post_L, residuals (in mm^3)
Grey Matter Volume of AAL region Parietal_Inf_R, residuals (in mm^3)
Grey Matter Volume of AAL region SupraMarginal_L, residuals (in mm^3)
Grey Matter Volume of AAL region SupraMarginal_R, residuals (in mm^3)
Grey Matter Volume of AAL region Angular_L, residuals (in mm^3)
Grey Matter Volume of AAL region Angular_R, residuals (in mm^3)
Grey Matter Volume of AAL region Precuneus_L, residuals (in mm^3)
Grey Matter Volume of AAL region Precuneus_R, residuals (in mm^3)
Grey Matter Volume of AAL region Temporal_Sup_L, residuals (in mm^3)
Grey Matter Volume of AAL region Temporal_Pole_Sup_L, residuals (in mm^3)
Grey Matter Volume of AAL region Temporal_Mid_R, residuals (in mm^3)
Grey Matter Volume of AAL region Cerebelum_10_L, residuals (in mm^3)

White-matter lesions and cerebral microbleeds: White matter lesions (WML) were assessed on FLAIR images using the ARWMC (age-related white-matter changes) scale (Xiong et al. 2010) across five regions (frontal, temporal, infratentorial, parieto-occipital, basal ganglia) in each hemisphere. Each region was scored on an ordinal scale from 0 to 3. The presence of WML was defined as an ARWMC score greater than 0 in any region. The total ARWMC (age-related WML) score was calculated as the

sum of all regional scores, ranging from 0 to 30. Interreader reliability assessment for WML was assessed in the context of WML volume quantification. All WML were segmented manually by a radiology resident with 2 years of neuroimaging experience. All segmentations were checked by a board-certified radiologist with 7 years neuroimaging experience and edited were necessary. Microbleeds were identified as small foci of signal loss (<2 mm) on T2*-weighted images and counted in the lobar, deep, and infratentorial regions of each hemisphere. Symmetric signal loss in the globus pallidum, likely due to calcification or vascular flow artifacts, as well as hemorrhagic intracerebral lesions, were excluded. [3-6]

Supplementary Material 2 – Inter- and intra-rater variability and intraclass correlation coefficients for the measurements of the signal intensity of the olfactory bulb

**Variable**	**Inter-rater variability (%)**	**Intraclass correlation coefficient**	**Intra-rater variability (%)**	**Intraclass correlation coefficient**
Right olfactory bulb - minimum	17 %	0.297	24%	0.340
Right olfactory bulb - mean	6 %	0.487	5%	0.647
Right olfactory bulb - maximum	4 %	0.466	30%	0.504
Left olfactory bulb - minimum	34%	0.267	25%	0.205
Left olfactory bulb - mean	6 %	0.376	1%	0.557
Left olfactory bulb - maximum	10 %	0.489	6%	0.592

Supplementary Material 3 – R Codes

Main model - example

```
#============================================================

# 1 - LOOP DOUBLE LOGISTIC BINOMIAL regression model function

#============================================================

logistic_bin_model <- function (thedat,

                                outVar,

                                confoundVar,

                                predVar,

                                PathTables,

                                PathTables_siglabel)


{

  theconfounders <- paste(confoundVar, collapse = " + ")


  # make results list
```

```
  reslist <- vector(mode ="list", length=length(outVar))
  names(reslist) <- outVar

  for (iOutcome in outVar){
    reslist[[iOutcome]] <- vector(mode ="list", length=length(predVar))
    names(reslist[[iOutcome]]) <- predVar

    for (iPred in predVar){
      # Model
      formul <- paste(iOutcome, "~", iPred, "+", theconfounders)
      themodel <- glm(formul, data = thedat, family = binomial, na.action = na.omit)

      # RESULTS:
      toSave <- as.data.frame(summary(themodel)$coefficients)
      reslist[[iOutcome]][[iPred]] <- toSave

      # SAVE to excel
      write.xlsx(toSave, file = file.path(PathTables, paste0("LogisticReg_", iOutcome, "_", iPred,
".xlsx")), rowNames = TRUE)

      # Significant results in extra foulder (--> SIGNIFICANT RESULTS 5%), with label, same for trend
      if (toSave[2, "Pr(>|z|)"] < 0.05){write.xlsx(toSave, file = file.path(PathTables_siglabel,
paste0("Sig_LogisticReg_", iOutcome, "_", iPred, ".xlsx")), rowNames= TRUE)}
      if ((toSave[2, "Pr(>|z|)"] < 0.1) & (toSave[2, "Pr(>|z|)"] >= 0.05)){write.xlsx(toSave, file =
file.path(PathTables_siglabel, paste0("Trend_LogisticReg_", iOutcome, "_", iPred, ".xlsx")), rowNames=
TRUE)}

    }
  }
  return(reslist)
}
#==============================================================
# 2 - LOOP DOUBLE linear regression model - function
#==============================================================
lin_model_loop <- function (thedat,
                            outVar,
                            confoundVar,
                            predVar,
                            PathTables,  #Path for all results
                            PathTables_siglabel) #Path with results sig. label
{
  theconfounders <- paste(confoundVar, collapse = " + ")
```

```
  # RESULTS List
  reslist <- vector(mode ="list", length=length(outVar))
  names(reslist) <- outVar

  for (iOutcome in outVar){

    reslist[[iOutcome]] <- vector(mode ="list", length=length(predVar))
    names(reslist[[iOutcome]]) <- predVar

    for (iPred in predVar){

      # MODEL
      formul <- paste(iOutcome, "~", iPred, "+", theconfounders)
      themodel <- lm(formul, data = thedat)

      # RESULTS to xlsx:
      toSave <- as.data.frame(summary(themodel)$coefficients)
      reslist[[iOutcome]][[iPred]] <- toSave
      write.xlsx(toSave, file = file.path(PathTables, paste0("LinReg_", iOutcome, "_", iPred,
".xlsx")), rowNames= TRUE)

      # significance label (--> SIGNIFICANT RESULTS 10%)
      if ((toSave[2, "Pr(>|t|)"] < 0.1) & (toSave[2, "Pr(>|t|)"] >= 0.05)){write.xlsx(toSave, file =
file.path(PathTables_siglabel, paste0("Trend_LinReg_", iOutcome, "_", iPred, ".xlsx")), rowNames=
TRUE)}

      # significance label (--> SIGNIFICANT RESULTS 5%)
      if (toSave[2, "Pr(>|t|)"] < 0.05){write.xlsx(toSave, file = file.path(PathTables_siglabel,
paste0("Sig_LinReg_", iOutcome, "_", iPred, ".xlsx")), rowNames= TRUE)}
    }
  }
  return(reslist)
}
#___________
# MODELS
#___________
#___________
# WML and Microbleeds
#___________
# Data
dat <- read.table("C:/mypath", header=TRUE, row.names = 1, sep = ",")

# Outcome
```

```
outcome.var <- c("wml_new", "myfrontallesion", "mynonfrontallesion", "myparietooclesion",
"mytemporallesion", "myinfratentoriallesion",
                 "mybasalganglesion", "mycerbralbleedings")
# Model
mylist <- logistic_bin_model(thedat  = dat,
                             outVar = outcome.var,
                             predVar = predictor.var,
                             confoundVar = confounder.var,
                             PathTables = file.path(path_model, "."),
                             PathTables_siglabel = file.path(path_model_sig, ".")
)
#___________
# Bulbus olfactorius
#___________
# Data
dat <- read.table("C:/mypath", header=TRUE, row.names = 1, sep = ",")
# Outcome
outcome.var <- c("bulbmean", "bulbmin", "bulbmax")
# Model
mylist <- lin_model_loop(thedat  = dat,
                         outVar = outcome.var,
                         predVar = predictor.var,
                         confoundVar = confounder.var,
                         PathTables = file.path(path_model, "."),
                         PathTables_siglabel = file.path(path_model_sig, ".")
)
#___________
# Brain volumes
#___________
# Data
dat <- read.table("C:/mypath", header=TRUE, row.names = 1, sep = ",")
# Outcome
outcome.var <- c("myhippoamygdalavol_icv", "my_gmearv_icv", "my_csfe_icv", "my_wme_icv",
                 "mybrainvol_icv")
# Model
mylist <- lin_model_loop(thedat  = dat,
                         outVar = outcome.var,
                         predVar = predictor.var,
                         confoundVar = confounder.var,
                         PathTables = file.path(path_model, "."),
                         PathTables_siglabel = file.path(path_model_sig, ".")
```

```
)
#__________
# Age-related WML
#__________
# Data
dat <- read.table("mypath", header=TRUE, row.names = 1, sep = ",")

# Outcome
outcome.var <- c("myNeuro_arwTOT")

# Model
mylist <- sqrtlin_model(thedat  = dat,
                        outVar = outcome.var,
                        predVar = predictor.var,
                        confoundVar = confounder.var,
                        PathTables = file.path(path_model, "."),
                        PathTables_siglabel = file.path(path_model_sig, ".")
)
```

Mediation analysis - example

```
#==============================================================
# 1 – Mediation
#==============================================================
#-------- PNC -----
#--- EXPOSURE - MEDIATOR
model.m <- lm(bulbmean ~  GC_PNC_14 + u3talteru + u3csex + u3tbmi + u3talkkon + u3tcigreg_sf + u3tphact
+ u3tfamstd + u3tedyrs ,
              data = dat,
              na.action = na.omit)
#summary(model.m)
#--- EXPOSURE - OUTCOME
model.y <- glm(wml_new ~ GC_PNC_14 + bulbmean + u3talteru + u3csex + u3tbmi + u3talkkon + u3tcigreg_sf
+ u3tphact + u3tfamstd + u3tedyrs ,
               data = dat,
               family = binomial,
               na.action = na.omit)
#summary(model.y)
#---  MEDIATION
med_model <- mediate(model.m, model.y, sims = 1000, boot = FALSE, treat = "GC_PNC_14",
                     mediator = "bulbmean")
summary(med_model)
```

SUPPLEMENTARY TABLES

Supplementary Table 1 – Study characteristics and environmental exposures by sex

(A) PARTICIPANT CHARACTERISTICS			
	**Women** n = 169	**Men** n = 231	**Test** p-value
	**Mean *(SD)***	**Mean *(SD)***	
**Age** [years]	56.3 *(9.0)*	56.3 *(9.3)*	0.970 ([a])
**Weight** [kg]	73.7 *(14.8)*	90.0 *(14.4)*	<0.001 ([a])
**BMI** [kg/m³]	27.7 *(5.5)*	28.5 *(4.4)*	0.11 ([a])
**Height** [cm]	163.3 *(6.5)*	177.7 *(11.3)*	<0.001 ([a])
**SBP** [mmHg]	113.0 *(14.5)*	126.2 *(16.2)*	<0.001 ([a])
**DBP** [mmHg]	72.0 *(8.5)*	77.7 *(10.3)*	<0.001 ([a])
**PP** [mmHg]	70.9 (7.7)	71.5 (11.3)	0.51 ([a])
**Cholesterol** [mg/dl]	219.8 *(34.9)*	216.6 *(37.4)*	0.39 ([a])
**HDL** [mg/dl]	70.1 *(17.3)*	55.8 *(15.1)*	<0.001 ([a])
**LDL** [mg/dl]	137.4 *(32.2)*	141.3 *(33.3)*	0.24 ([a])
**TAG** [mg/dl]	103.0 *(45.8)*	153.0 *(99.4)*	<0.001 ([a])
	**Median *(IQR)***	**Median *(IQR)***	
**Alcohol consumption** [g/day]	2.9 *(12.3)*	20.0 *(16.4)*	<0.001 ([b])
**hsCRP** [mg/l]	1.4 *(2.1)*	1.1 *(1.7)*	0.07 ([b])
	**N *(%)***	**N *(%)***	
**Diabetes status**			0.002 ([c])
Diabetes	14 *(8%)*	40 *(17%)*	
Prediabetes	37 *(22%)*	66 *(29%)*	
Normoglycemia	118 *(70%)*	125 *(54%)*	
**Antihypertensive medication**	47 *(28%)*	55 *(24%)*	0.423 ([c])
**Lipid lowering medication**	17 *(10%)*	25 *(11%)*	0.940 ([c])
**Antidiabetic medication**	12 *(7%)*	20 *(9%)*	0.704 ([c])
**Household income per month**			0.615 ([d])
<625€	7 *(4%)*	7 *(3%)*	
625€ to <1250€	45 *(27%)*	61 *(26%)*	
1250€ to <1875€	79 *(47%)*	113 *(49%)*	
1875€ to <2500€	6 *(4%)*	5 *(2%)*	
≥2500€	20 *(12%)*	39 *(17%)*	
missing	12 *(5%)*	6 *(3%)*	
**Marital status**			<0.001 ([d])
Unmarried, living alone	15 *(8%)*	24 *(10%)*	
Unmarried, living with the partner	8 *(5%)*	7 *(3%)*	

Married, living with the spouse	107 *(65%)*	182 *(79%)*	
Married, living apart	5 *(3%)*	4 *(2%)*	
Divorced	19 *(11%)*	12 *(5%)*	
Widowed	15 *(8%)*	2 *(1%)*	
**Years of education**			<0.001 ([d])
8	6 *(4%)*	4 *(2%)*	
10	71 *(42%)*	66 *(29%)*	
11	31 *(18%)*	22 *(10%)*	
12	11 *(7%)*	27 *(11%)*	
13	31 *(18%)*	49 *(21%)*	
15	0 *(0%)*	5 *(2%)*	
17	19 *(11%)*	58 *(25%)*	
**Smoking habits**			0.079 ([c])
Regular	35 *(21%)*	45 *(20%)*	
Former	63 *(38%)*	111 *(48%)*	
Never	70 *(42%)*	74 *(32%)*	
**Physical activity**			0.107 ([c])
Very active	47 *(28%)*	68 *(30%)*	
Moderate active	61 *(36%)*	62 *(27%)*	
Little active	25 *(15%)*	31 *(13%)*	
Non-active	35 *21%)*	69 *(30%)*	
**(B) ENVIRONMENTAL EXPOSURE**			
**PM_{10}** [μg/m^3]	16.5 *(2.1)*	16.4 *(2.0)*	0.516 ([a])
**$PM_{2.5}$** [μg/m^3]	11.7 *(1.4)*	11.7 *(1.4)*	0.776 ([a])
**PM_{coarse}** [μg/m^3]	4.8 *(1.3)*	4.8 *(1.5)*	0.566 ([a])
**PNC** [n/m^3]	7,045.2 *(2,140.1)*	7,124.1 *(2,320.1)*	0.695 ([a])
**NO_2** [μg/m^3]	13.6 *(6.1)*	13.7 *(5.8)*	0.866 ([a])
**NO_X** [μg/m^3]	20.8 *(9.6)*	21.4 *(9.5)*	0.439 ([a])
**$PM_{2.5}$abs** [10^{-5} m^{-1}]	1.2 *(0.3)*	1.2 *(0.3)*	0.787 ([a])
**(C) CRANIAL MRI PARAMETER**			
**Global** WML	108 (63.9)	141 (61.0)	0.448 ([c])
**Frontal** WML	103 (60.9)	135 (58.4)	0.456 ([c])
**Temporal** WML	63 (37.3)	81 (35.1)	0.450 ([c])
**Parieto-occipital** WML	69 (40.8)	76 (32.9)	0.157 ([c])
**Infratentorial** WML	4 (2.4)	10 (4.3)	0.258 ([d])
**Non-frontal** WML	81 (47.9)	106 (45.9)	0.461 ([c])
**Basal ganglia** WML	28 (16.6)	38 (16.5)	0.469 ([c])
**Cerebral microbleedings**	13 (7.7)	21 (9.1)	0.233 ([c])

**Age-related** WML	3.16 (3.46)	3.07 (3.70)	0.797 ([a])
**ICV**	0.83 (0.03)	0.83 (0.03)	0.670 ([a])
**GM volume** (all reliable)	0.08 (0.00)	0.08 (0.00)	0.896 ([a])
**WM volume**	0.41 (0.01)	0.41 (0.02)	0.342 ([a])
**CSF volume**	0.17 (0.03)	0.17 (0.03)	0.670 ([a])
**Hippocampus and Amygdala volume**	0.01 (0.00)	0.01 (0.00)	0.002 ([a])
**Olfactory bulb mean**	7.42 (2.71)	7.83 (3.28)	0.236 ([a])
**Olfactory bulb minimum**	5.96 (2.21)	6.42 (2.82)	0.119 ([a])
**Olfactory bulb maximum**	8.50 (3.11)	8.93 (3.73)	0.284 ([a])

Table 1: Participant characteristics and environmental exposure according to sex. SD: standard deviation; IQR: interquartile range; SBP: systolic blood pressure in mmHg; DBP: diastolic blood pressure in mmHg; PP: pulse pressure in mmHg; BMI: Body mass index; WHR: Waist-to-hip ratio; HDL: High densitiy lipoprotein; LDL: Low density lipoprotein; TAG: Triacylglycerides; hsCRP: high sensitive c-reactive protein. PM_{10}: particulate matter with an aerodynamic diameter ≥ 10µm. $PM_{2.5}$: particulate matter with an aerodynamic diameter ≥ 2.5µm. PM_{coarse}: particles with an aerodynamic parameter 10-2.5 µm. PNC: particle number concentration. NO_2: nitrogen dioxide. NO_X: nitrogen oxides. PM25abs: $PM_{2.5}$ absorbance; WML: white-matter lesions; ICV: intracranial volume; GM: grey matter; WM: white matter; CSF: cerebro-spinal fluid. t-Test: (a); Wilcoxon-Rank-Test for non-normal distributed variables: (b); Chi-Square-Test: (c); Fishers Exact Test: (d).

Supplementary Table 2 – Air pollutant's correlation matrix

	**PM_{10}**	**$PM_{2.5}$**	**PM_{Coarse}**	**PNC**	**NO_2**	**NO_X**	**$PM_{2.5}$abs**
**PM_{10}**	1	0.50	0.79	0.82	0.76	0.75	0.80
**$PM_{2.5}$**		1	0.53	0.62	0.70	0.75	0.60
**PM_{Coarse}**			1	0.76	0.81	0.73	0.77
**PNC**				1	0.78	0.93	0.76
**NO_2**					1	0.85	0.86
**NO_X**						1	0.73
**$PM_{2.5}$abs**							1

Table 2: Pearson correlation coefficients. PM_{10}: particulate matter with an aerodynamic diameter ≥ 10µm. $PM_{2.5}$: particulate matter with an aerodynamic diameter ≥ 2.5µm. PM_{coarse}: particles with an aerodynamic parameter 10-2.5 µm. PNC: particle number concentration. NO_2: nitrogen dioxide. NO_X: nitrogen oxides. $PM_{2.5}$abs: $PM_{2.5}$ absorbance

Supplementary Table 3 – Association between TRAP and brain morphology

Outcome	Model	PNC	NO_2	NO_X	$PM_{2.5}$abs	PM_{10}	$PM_{2.5}$	PM_{coarse}
**Global WML**	Model_1	1.2 [0.92, 1.57]	1.15 [0.83, 1.59]	1.14 [0.85, 1.52]	1.38 [0.96, 1.97]	1.25 [0.9, 1.74]	1.13 [0.82, 1.56]	1.23 [0.89, 1.71]
**Global WML**	Model_3	1.3 [0.98, 1.72]	1.29 [0.92, 1.81]	1.24 [0.91, 1.68]	1.48 [1.02, 2.14]	1.34 [0.94, 1.89]	1.24 [0.89, 1.74]	1.36 [0.96, 1.92]
**Frontal WML**	Model_1	1.12 [0.86, 1.46]	1.03 [0.75, 1.42]	1.05 [0.78, 1.39]	1.26 [0.89, 1.79]	1.2 [0.87, 1.65]	1.03 [0.75, 1.41]	1.15 [0.83, 1.58]
**Frontal WML**	Model_3	1.2 [0.91, 1.58]	1.14 [0.82, 1.59]	1.12 [0.83, 1.51]	1.36 [0.95, 1.95]	1.28 [0.91, 1.79]	1.11 [0.8, 1.54]	1.27 [0.9, 1.77]
**Non-frontal WML**	Model_1	1.18 [0.92, 1.53]	1.28 [0.94, 1.75]	1.18 [0.89, 1.57]	1.48 [1.05, 2.09]	1.32 [0.96, 1.8]	1.25 [0.92, 1.7]	1.48 [1.08, 2.04]
**Non-frontal WML**	Model_3	1.25 [0.96, 1.63]	1.42 [1.03, 1.96]	1.26 [0.94, 1.69]	1.57 [1.1, 2.23]	1.38 [1, 1.92]	1.37 [1, 1.89]	1.61 [1.15, 2.25]
**Parieto-occipital WML**	Model_1	1.1 [0.85, 1.43]	1.13 [0.83, 1.54]	1.09 [0.82, 1.45]	1.14 [0.82, 1.61]	1.17 [0.86, 1.6]	1.06 [0.77, 1.45]	1.29 [0.94, 1.78]
**Parieto-occipital WML**	Model_3	1.18 [0.9, 1.54]	1.27 [0.92, 1.76]	1.17 [0.87, 1.57]	1.25 [0.88, 1.78]	1.26 [0.91, 1.75]	1.16 [0.84, 1.61]	1.45 [1.03, 2.02]
**Temporal WML**	Model_1	1.04 [0.8, 1.34]	1.13 [0.83, 1.55]	1.06 [0.79, 1.41]	1.24 [0.88, 1.74]	1.05 [0.77, 1.44]	1.18 [0.86, 1.62]	1.15 [0.84, 1.58]
**Temporal WML**	Model_3	1.11 [0.85, 1.45]	1.26 [0.91, 1.75]	1.14 [0.85, 1.54]	1.36 [0.95, 1.94]	1.12 [0.81, 1.56]	1.32 [0.95, 1.83]	1.26 [0.9, 1.76]
**Basal ganglia WML**	Model_1	1.22 [0.89, 1.67]	1.29 [0.88, 1.88]	1.26 [0.88, 1.81]	1.39 [0.92, 2.1]	1.12 [0.76, 1.64]	1.14 [0.77, 1.68]	1.4 [0.95, 2.06]
**Basal ganglia WML**	Model_3	1.26 [0.92, 1.74]	1.35 [0.91, 1.99]	1.32 [0.91, 1.9]	1.48 [0.96, 2.29]	1.17 [0.79, 1.73]	1.2 [0.81, 1.8]	1.47 [0.98, 2.2]
**Microbleeds**	Model_1	1.18 [0.79, 1.77]	1.14 [0.7, 1.85]	1.23 [0.77, 1.96]	1.23 [0.72, 2.09]	1.21 [0.75, 1.98]	1.08 [0.65, 1.77]	1.11 [0.67, 1.82]
**Microbleeds**	Model_3	1.27 [0.82, 1.97]	1.18 [0.69, 2]	1.32 [0.8, 2.18]	1.46 [0.8, 2.65]	1.36 [0.79, 2.32]	1.17 [0.69, 1.99]	1.16 [0.67, 2.02]
**Intracranial volume**	Model_1	0.06 [-0.29, 0.42]	0.04 [-0.38, 0.46]	-0.06 [-0.45, 0.33]	-0.12 [-0.57, 0.33]	-0.1 [-0.53, 0.33]	-0.24 [-0.66, 0.17]	0.18 [-0.26, 0.61]
**Intracranial volume**	Model_3	0.06 [-0.29, 0.42]	0.02 [-0.4, 0.45]	-0.04 [-0.44, 0.35]	-0.15 [-0.61, 0.3]	-0.12 [-0.56, 0.31]	-0.23 [-0.66, 0.19]	0.13 [-0.31, 0.57]
**Grey-matter volume**	Model_1	0.02 [-0.62, 0.66]	-0.2 [-0.96, 0.56]	-0.23 [-0.94, 0.48]	-0.21 [-1.03, 0.6]	-0.4 [-1.17, 0.38]	-0.46 [-1.22, 0.29]	0.14 [-0.65, 0.92]
**Grey-matter volume**	Model_3	-0.03 [-0.68, 0.61]	-0.27 [-1.04, 0.5]	-0.26 [-0.97, 0.45]	-0.37 [-1.19, 0.45]	-0.5 [-1.28, 0.28]	-0.5 [-1.26, 0.27]	0 [-0.8, 0.8]
**White-matter volume**	Model_1	0.1 [-0.35, 0.55]	0.04 [-0.5, 0.58]	-0.02 [-0.52, 0.48]	-0.18 [-0.75, 0.39]	-0.08 [-0.62, 0.47]	-0.3 [-0.83, 0.23]	0.19 [-0.36, 0.75]
**White-matter volume**	Model_3	0.12 [-0.33, 0.58]	0.05 [-0.49, 0.59]	0.03 [-0.47, 0.53]	-0.2 [-0.78, 0.38]	-0.1 [-0.65, 0.45]	-0.28 [-0.82, 0.26]	0.17 [-0.4, 0.73]
**Cerebro-spinal fluid volume**	Model_1	-0.31 [-2.02, 1.41]	-0.19 [-2.23, 1.86]	0.29 [-1.6, 2.19]	0.57 [-1.61, 2.74]	0.48 [-1.6, 2.56]	1.18 [-0.84, 3.21]	-0.85 [-2.95, 1.25]
**Cerebro-spinal fluid volume**	Model_3	-0.31 [-2.04, 1.42]	-0.11 [-2.18, 1.95]	0.2 [-1.71, 2.11]	0.74 [-1.47, 2.95]	0.6 [-1.5, 2.7]	1.13 [-0.92, 3.18]	-0.62 [-2.76, 1.52]
**Hippocampus/Amygdala volume**	Model_1	0.22 [-0.6, 1.03]	0.04 [-0.93, 1.01]	-0.02 [-0.92, 0.89]	-0.3 [-1.33, 0.74]	-0.17 [-1.16, 0.82]	-0.45 [-1.41, 0.51]	0.66 [-0.34, 1.65]

**Hippocampus/Amygdala volume**	Model_3	0.22 [-0.6, 1.04]	-0.02 [-0.99, 0.96]	0.05 [-0.86, 0.95]	-0.4 [-1.44, 0.65]	-0.28 [-1.28, 0.71]	-0.39 [-1.36, 0.59]	0.57 [-0.44, 1.59]
**Olfactory bulb mean**	Model_1	0.43 [-4.72, 5.58]	-1.96 [-8.18, 4.27]	-0.1 [-5.81, 5.62]	-4.37 [-10.95, 2.21]	-4.03 [-10.31, 2.25]	-4.95 [-11.27, 1.37]	-1.8 [-8.15, 4.56]
**Olfactory bulb mean**	Model_3	1.03 [-4.16, 6.23]	-1.36 [-7.64, 4.93]	0.21 [-5.57, 5.99]	-3.36 [-10.01, 3.29]	-3.38 [-9.74, 2.98]	-4.86 [-11.26, 1.54]	-0.62 [-7.07, 5.83]
**Olfactory bulb maximum**	Model_1	0.42 [-4.72, 5.57]	-1.89 [-8.11, 4.33]	0.02 [-5.69, 5.74]	-4.38 [-10.96, 2.2]	-4.18 [-10.46, 2.1]	-4.91 [-11.23, 1.41]	-1.62 [-7.97, 4.73]
**Olfactory bulb maximum**	Model_3	1.03 [-4.16, 6.23]	-1.33 [-7.61, 4.95]	0.33 [-5.45, 6.11]	-3.41 [-10.06, 3.23]	-3.46 [-9.82, 2.89]	-4.79 [-11.19, 1.61]	-0.43 [-6.87, 6.02]

Table 3: Associations between TRAP and brain morphology. Results are presented as OR (WML, microbleeds) or %-changes (volumes) and 95%-confidence intervals for brain volumes per interquartile-range (IQR) increase in the respective air pollutant. Model 1 was adjusted for age, sex. Model 3 was adjusted for age, sex, BMI, alcohol consumption, smoking, physical activity, marital status and education years.

Supplementary Table 4 – Sensitivity analysis adjusting for hs-CRP

Outcome	**PNC**	**NO_2**	**NO_X**	**$PM_{2.5}$abs**	**PM_{10}**	**$PM_{2.5}$**	**PM_{coarse}**
Global WML	1.32 [0.99, 1.75]	1.35 [0.95, 1.9]	1.27 [0.93, 1.72]	1.57 [1.08, 2.29]	1.39 [0.98, 1.97]	1.31 [0.93, 1.85]	1.41 [0.99, 2]
Frontal WML	1.22 [0.93, 1.6]	1.19 [0.85, 1.66]	1.14 [0.85, 1.55]	1.44 [0.99, 2.07]	1.33 [0.94, 1.87]	1.16 [0.83, 1.63]	1.3 [0.92, 1.84]
Non-frontal WML	1.27 [0.97, 1.66]	1.49 [1.07, 2.07]	1.3 [0.97, 1.75]	1.67 [1.16, 2.41]	1.42 [1.02, 1.97]	1.48 [1.06, 2.06]	1.63 [1.16, 2.3]
Parieto-occipital WML	1.19 [0.91, 1.56]	1.33 [0.95, 1.85]	1.21 [0.9, 1.63]	1.33 [0.92, 1.91]	1.28 [0.92, 1.79]	1.25 [0.89, 1.74]	1.46 [1.04, 2.06]
Temporal WML	1.11 [0.85, 1.46]	1.29 [0.93, 1.79]	1.15 [0.86, 1.55]	1.4 [0.98, 2.02]	1.13 [0.81, 1.58]	1.36 [0.97, 1.89]	1.26 [0.9, 1.77]
Basal ganglia WML	1.28 [0.92, 1.77]	1.41 [0.95, 2.1]	1.36 [0.94, 1.98]	1.61 [1.03, 2.52]	1.18 [0.79, 1.77]	1.3 [0.86, 1.95]	1.47 [0.97, 2.23]
Age-related WML	0.1 [-0.02, 0.22]	0.12 [-0.03, 0.27]	0.08 [-0.05, 0.21]	0.2 [0.04, 0.36]	0.12 [-0.03, 0.27]	0.08 [-0.07, 0.23]	0.16 [0.01, 0.31]
Microbleeds	1.27 [0.82, 1.98]	1.18 [0.69, 2]	1.33 [0.81, 2.19]	1.46 [0.79, 2.68]	1.36 [0.8, 2.31]	1.18 [0.68, 2.03]	1.19 [0.68, 2.08]
Intracranial volume	0.05 [-0.31, 0.4]	-0.04 [-0.47, 0.38]	-0.08 [-0.47, 0.31]	-0.22 [-0.67, 0.24]	-0.14 [-0.57, 0.29]	-0.3 [-0.73, 0.12]	0.09 [-0.36, 0.53]
Grey-matter volume	-0.05 [-0.69, 0.6]	-0.3 [-1.07, 0.47]	-0.29 [-1, 0.43]	-0.4 [-1.23, 0.44]	-0.5 [-1.28, 0.28]	-0.55 [-1.32, 0.22]	-0.03 [-0.84, 0.77]
White-matter volume	0.11 [-0.34, 0.55]	-0.03 [-0.57, 0.51]	-0.02 [-0.51, 0.48]	-0.27 [-0.85, 0.31]	-0.12 [-0.67, 0.42]	-0.36 [-0.89, 0.17]	0.12 [-0.44, 0.68]
Cerebro-spinal fluid volume	-0.23 [-1.94, 1.48]	0.2 [-1.85, 2.25]	0.39 [-1.5, 2.27]	1.04 [-1.17, 3.25]	0.7 [-1.38, 2.77]	1.47 [-0.57, 3.51]	-0.41 [-2.55, 1.73]
Hippocampus/Amygdala volume	0.22 [-0.6, 1.04]	0 [-0.99, 0.98]	0.05 [-0.86, 0.96]	-0.38 [-1.44, 0.69]	-0.26 [-1.26, 0.74]	-0.37 [-1.36, 0.61]	0.58 [-0.45, 1.61]

Table 4: Sensitivity analysis of the association between TRAP and brain morphology, additionally adjusting for hs-CRP. Results are presented as OR (WML, microbleeds), regression coefficients (age-related WML), or %-changes (volumes) and 95%-confidence intervals for brain volumes per interquartile-range (IQR) increase in the respective air pollutant. In addition to the variables specific to each sensitivity analysis, the model. was adjusted for age, sex, BMI, alcohol consumption, smoking, physical activity, marital status and education years.

Supplementary Table 5 – Sensitivity analysis adjusting for ICV

Outcome	**PNC**	**NO_2**	**NO_X**	**$PM_{2.5}abs$**	**PM_{10}**	**$PM_{2.5}$**	**PM_{coarse}**
Global WML	1.28 [0.94, 1.74]	1.25 [0.87, 1.81]	1.25 [0.9, 1.75]	1.4 [0.93, 2.1]	1.29 [0.88, 1.89]	1.19 [0.83, 1.71]	1.5 [1.01, 2.21]
Frontal WML	1.18 [0.88, 1.6]	1.14 [0.79, 1.62]	1.14 [0.82, 1.57]	1.31 [0.89, 1.95]	1.23 [0.85, 1.79]	1.09 [0.77, 1.56]	1.4 [0.96, 2.05]
Non-frontal WML	1.31 [0.98, 1.76]	1.44 [1.02, 2.05]	1.37 [0.99, 1.88]	1.57 [1.07, 2.3]	1.38 [0.96, 1.99]	1.41 [1, 1.99]	1.76 [1.21, 2.56]
Parieto-occipital WML	1.2 [0.89, 1.61]	1.31 [0.92, 1.85]	1.21 [0.88, 1.66]	1.33 [0.9, 1.95]	1.24 [0.87, 1.78]	1.22 [0.86, 1.72]	1.54 [1.07, 2.24]
Temporal WML	1.13 [0.83, 1.52]	1.27 [0.89, 1.8]	1.2 [0.87, 1.66]	1.35 [0.92, 1.99]	1.1 [0.76, 1.58]	1.36 [0.96, 1.93]	1.32 [0.91, 1.91]
Basal ganglia WML	1.19 [0.83, 1.7]	1.27 [0.84, 1.94]	1.23 [0.82, 1.83]	1.42 [0.89, 2.28]	1.06 [0.68, 1.64]	1.09 [0.72, 1.65]	1.48 [0.95, 2.31]
Age-related WML	0.1 [-0.03, 0.23]	0.11 [-0.04, 0.27]	0.1 [-0.04, 0.24]	0.18 [0.01, 0.35]	0.11 [-0.05, 0.27]	0.08 [-0.08, 0.24]	0.19 [0.03, 0.36]
Microbleeds	1.3 [0.81, 2.08]	1.11 [0.63, 1.96]	1.33 [0.78, 2.28]	1.35 [0.69, 2.64]	1.4 [0.78, 2.52]	1.05 [0.6, 1.83]	1.24 [0.68, 2.26]

Table 5: Sensitivity analysis of the association between TRAP and brain morphology, additionally adjusting for intracranial volume (ICV). Results are presented as OR (WML, microbleeds), regression coefficients (age-related WML), or %-changes (volumes) and 95%-confidence intervals for brain volumes per interquartile-range (IQR) increase in the respective air pollutant. In addition to the variables specific to each sensitivity analysis, the model. was adjusted for age, sex, BMI, alcohol consumption, smoking, physical activity, marital status and education years.

Supplementary Table 6 – Sensitivity analysis adjusting for degree of urbanisation

Outcome	PNC	NO_2	NO_X	$PM_{2.5}abs$	PM_{10}	$PM_{2.5}$	PM_{coarse}
**Global WML**	1.32 [0.97, 1.81]	1.35 [0.88, 2.06]	1.23 [0.87, 1.74]	1.67 [1.04, 2.69]	1.35 [0.93, 1.96]	1.21 [0.84, 1.75]	1.44 [0.94, 2.2]
**Frontal WML**	1.24 [0.91, 1.68]	1.19 [0.78, 1.81]	1.13 [0.8, 1.58]	1.57 [0.99, 2.51]	1.32 [0.92, 1.91]	1.09 [0.76, 1.57]	1.37 [0.91, 2.08]
**Non-frontal WML**	1.19 [0.88, 1.6]	1.41 [0.93, 2.13]	1.19 [0.85, 1.66]	1.62 [1.02, 2.57]	1.33 [0.93, 1.9]	1.3 [0.91, 1.84]	1.67 [1.1, 2.53]
**Parieto-occipital WML**	1.17 [0.86, 1.58]	1.33 [0.87, 2.02]	1.15 [0.82, 1.61]	1.26 [0.79, 2.02]	1.26 [0.88, 1.82]	1.12 [0.78, 1.6]	1.57 [1.03, 2.4]
**Temporal WML**	1.06 [0.78, 1.44]	1.25 [0.82, 1.9]	1.08 [0.77, 1.52]	1.38 [0.86, 2.21]	1.07 [0.74, 1.55]	1.27 [0.89, 1.83]	1.22 [0.8, 1.86]
**Basal ganglia WML**	1.26 [0.88, 1.81]	1.43 [0.85, 2.38]	1.32 [0.87, 2.02]	1.72 [0.96, 3.09]	1.12 [0.72, 1.74]	1.17 [0.75, 1.81]	1.63 [0.98, 2.72]
**Age-related WML**	0.08 [-0.06, 0.22]	0.08 [-0.11, 0.27]	0.04 [-0.11, 0.2]	0.19 [-0.02, 0.41]	0.09 [-0.07, 0.26]	0.02 [-0.15, 0.18]	0.16 [-0.03, 0.35]
**Microbleeds**	1.22 [0.73, 2.01]	1.03 [0.52, 2.07]	1.27 [0.72, 2.26]	1.52 [0.67, 3.43]	1.28 [0.7, 2.34]	1.09 [0.61, 1.95]	1.04 [0.51, 2.1]
**Intracranial volume**	-0.02 [-0.42, 0.39]	-0.17 [-0.72, 0.37]	-0.17 [-0.62, 0.29]	-0.45 [-1.05, 0.16]	-0.27 [-0.75, 0.21]	-0.38 [-0.85, 0.08]	0.06 [-0.49, 0.62]
**Grey-matter volume**	-0.22 [-0.95, 0.5]	-0.8 [-1.76, 0.16]	-0.55 [-1.35, 0.26]	-1.07 [-2.15, 0]	-0.79 [-1.64, 0.07]	-0.78 [-1.61, 0.04]	-0.33 [-1.32, 0.65]
**White-matter volume**	0.07 [-0.45, 0.59]	-0.1 [-0.79, 0.59]	-0.06 [-0.63, 0.52]	-0.48 [-1.25, 0.29]	-0.23 [-0.85, 0.38]	-0.4 [-1, 0.19]	0.15 [-0.55, 0.86]
**Cerebro-spinal fluid volume**	0.08 [-1.89, 2.04]	0.84 [-1.78, 3.45]	0.8 [-1.38, 2.99]	2.16 [-0.75, 5.08]	1.29 [-1.03, 3.6]	1.86 [-0.38, 4.09]	-0.31 [-2.98, 2.36]
**Hippocampus/Amygdala volume**	0.2 [-0.72, 1.13]	-0.21 [-1.44, 1.02]	-0.04 [-1.07, 0.99]	-0.88 [-2.25, 0.5]	-0.43 [-1.53, 0.66]	-0.58 [-1.63, 0.48]	0.74 [-0.51, 2]

Table 6: Sensitivity analysis of the association between TRAP and brain morphology, additionally adjusting for degree of urbanisation. Results are presented as OR (WML, microbleeds), regression coefficients (age-related WML), or %-changes (volumes) and 95%-confidence intervals for brain volumes per interquartile-range (IQR) increase in the respective air pollutant. In addition to the variables specific to each sensitivity analysis, the model. was adjusted for age, sex, BMI, alcohol consumption, smoking, physical activity, marital status and education years.

Supplementary Table 7 – Sensitivity analysis excluding movers

Outcome	PNC	NO_2	NO_X	$PM_{2.5}abs$	PM_{10}	$PM_{2.5}$	PM_{coarse}
**Global WML**	1.3 [0.98, 1.73]	1.23 [0.88, 1.74]	1.22 [0.9, 1.66]	1.4 [0.96, 2.04]	1.31 [0.92, 1.85]	1.22 [0.86, 1.73]	1.31 [0.92, 1.86]
**Frontal WML**	1.21 [0.92, 1.6]	1.11 [0.8, 1.55]	1.12 [0.83, 1.51]	1.31 [0.9, 1.89]	1.25 [0.89, 1.76]	1.12 [0.8, 1.57]	1.24 [0.88, 1.74]
**Non-frontal WML**	1.25 [0.95, 1.64]	1.35 [0.97, 1.87]	1.24 [0.92, 1.67]	1.45 [1.01, 2.08]	1.34 [0.96, 1.87]	1.31 [0.94, 1.82]	1.53 [1.09, 2.15]
**Parieto-occipital WML**	1.17 [0.89, 1.53]	1.15 [0.83, 1.6]	1.13 [0.83, 1.52]	1.12 [0.78, 1.61]	1.21 [0.87, 1.68]	1.06 [0.76, 1.48]	1.37 [0.97, 1.92]
**Temporal WML**	1.08 [0.82, 1.42]	1.17 [0.84, 1.63]	1.09 [0.81, 1.47]	1.22 [0.85, 1.76]	1.07 [0.76, 1.5]	1.27 [0.91, 1.78]	1.17 [0.83, 1.65]
**Basal ganglia WML**	1.35 [0.98, 1.87]	1.37 [0.93, 2.04]	1.41 [0.97, 2.04]	1.55 [1, 2.42]	1.19 [0.8, 1.78]	1.27 [0.84, 1.92]	1.49 [0.99, 2.25]
**Age-related WML**	0.1 [-0.02, 0.23]	0.08 [-0.07, 0.23]	0.07 [-0.07, 0.21]	0.15 [-0.02, 0.31]	0.11 [-0.05, 0.26]	0.05 [-0.11, 0.2]	0.14 [-0.02, 0.29]
**Microbleeds**	1.22 [0.78, 1.91]	1.07 [0.63, 1.84]	1.28 [0.78, 2.12]	1.33 [0.73, 2.45]	1.25 [0.72, 2.16]	1.14 [0.66, 1.97]	1.11 [0.63, 1.96]
**Intracranial volume**	0.07 [-0.3, 0.44]	0.02 [-0.42, 0.46]	-0.03 [-0.44, 0.37]	-0.06 [-0.54, 0.42]	-0.13 [-0.58, 0.31]	-0.23 [-0.68, 0.21]	0.15 [-0.3, 0.61]
**Grey-matter volume**	-0.06 [-0.72, 0.61]	-0.32 [-1.11, 0.47]	-0.3 [-1.03, 0.43]	-0.37 [-1.22, 0.48]	-0.54 [-1.34, 0.26]	-0.54 [-1.34, 0.26]	-0.02 [-0.84, 0.8]
**White-matter volume**	0.14 [-0.33, 0.61]	0.07 [-0.49, 0.63]	0.06 [-0.46, 0.58]	-0.09 [-0.7, 0.51]	-0.11 [-0.68, 0.47]	-0.26 [-0.83, 0.31]	0.19 [-0.39, 0.78]
**Cerebro-spinal fluid volume**	-0.34 [-2.12, 1.44]	-0.1 [-2.23, 2.04]	0.16 [-1.8, 2.13]	0.29 [-2.01, 2.58]	0.65 [-1.51, 2.81]	1.12 [-1.03, 3.27]	-0.75 [-2.95, 1.46]
**Hippocampus/Amygdala volume**	0.27 [-0.58, 1.12]	-0.01 [-1.02, 1]	0.08 [-0.85, 1.01]	-0.29 [-1.38, 0.79]	-0.25 [-1.28, 0.77]	-0.34 [-1.36, 0.68]	0.56 [-0.48, 1.61]

Table 7: Sensitivity analysis of the association between TRAP and brain morphology, excluding movers. Results are presented as OR (WML, microbleeds), regression coefficients (age-related WML), or %-changes (volumes) and 95%-confidence intervals for brain volumes per interquartile-range (IQR) increase in the respective air pollutant. In addition to the variables specific to each sensitivity analysis, the model. was adjusted for age, sex, BMI, alcohol consumption, smoking, physical activity, marital status and education years.

<u>SUPPLEMENTARY FIGURES</u>

Supplementary Figure 1 – Association between TRAP and white-matter lesions and cerebral microbleeds, mediated by the mean signal intensity of the olfactory bulb

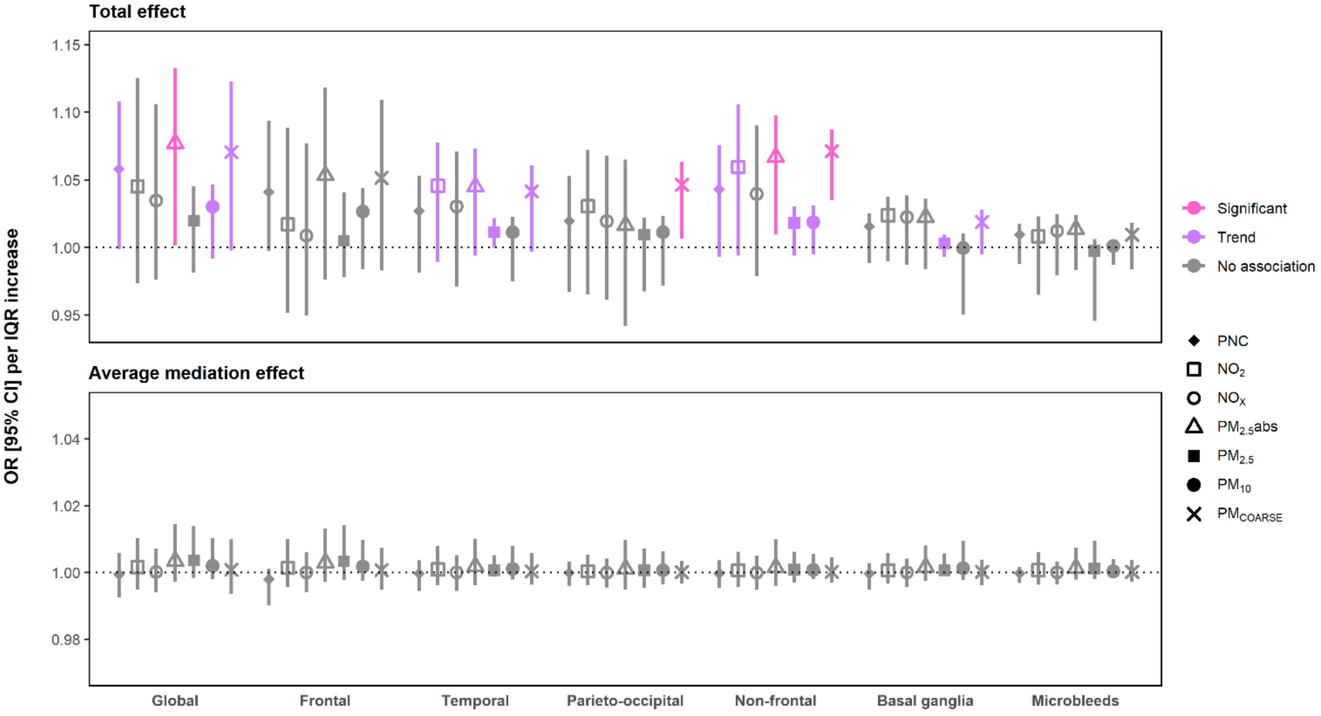

Figure 1: Associations between TRAP and white-matter lesions and cerebral microbleeds, mediated by the mean signal intensity of the olfactory bulb. The upper panel presents odds ratios and 95%-confidence intervals of the total effect per interquartile-range (IQR) increase in the respective air pollutant. The lower panel presents odds ratios and 95%-confidence intervals of the average mediation effect per IQR increase in the respective air pollutant. Strength of association derived from the mediation analysis: significant: p-value < 0.05. Trend: 0.1 > p-value ≥ 0.05. No association: p-value ≥ 0.1. Models were adjusted for age, sex, BMI, alcohol consumption, smoking, physical activity, marital status and education years. N = 322.

Supplementary Figure 2 – Association between TRAP and white-matter lesions and cerebral microbleeds, mediated by the maximum signal intensity of the olfactory bulb

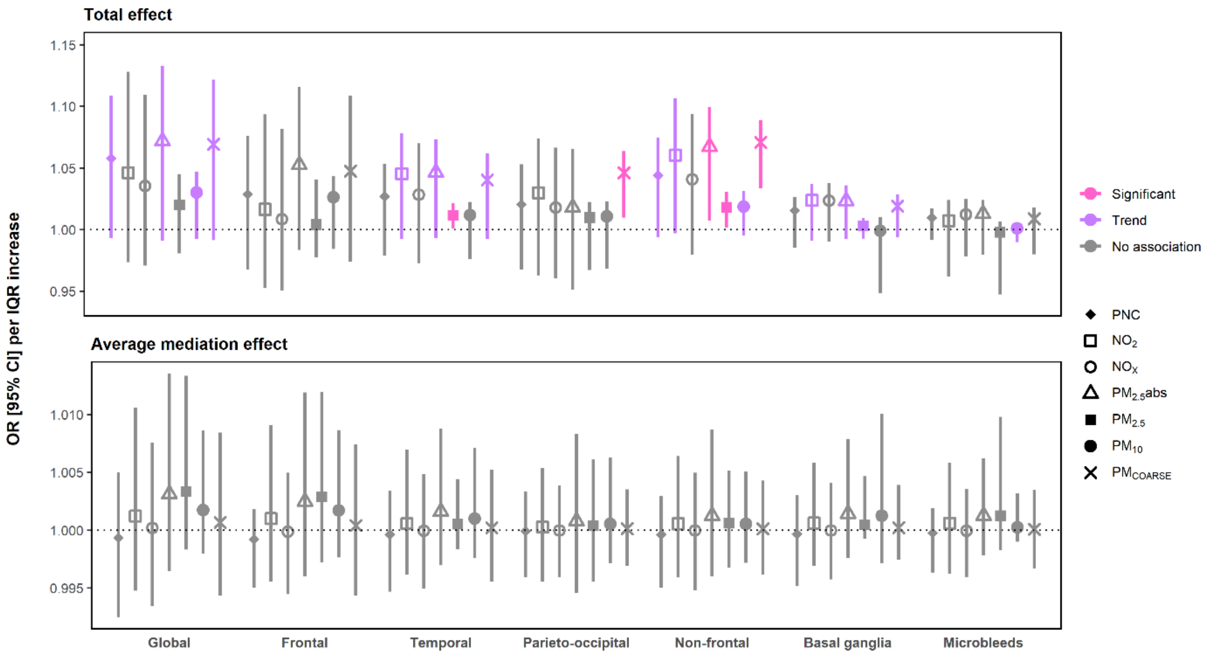

Figure 2: Associations between TRAP and white-matter lesions and cerebral microbleeds, mediated by the maximum signal intensity of the olfactory bulb. The upper panel presents odds ratios and 95%-confidence intervals of the total effect per interquartile-range (IQR) increase in the respective air pollutant. The lower panel presents odds ratios and 95%-confidence intervals of the average mediation effect per IQR increase in the respective air pollutant. Strength of association derived from the mediation analysis: significant: p-value < 0.05. Trend: 0.1 > p-value ≥ 0.05. No association: p-value ≥ 0.1. Models were adjusted for age, sex, BMI, alcohol consumption, smoking, physical activity, marital status and education years. N = 322.

Supplementary Figure 3 – Association between TRAP and brain volumes, mediated by the mean signal intensity of the olfactory bulb

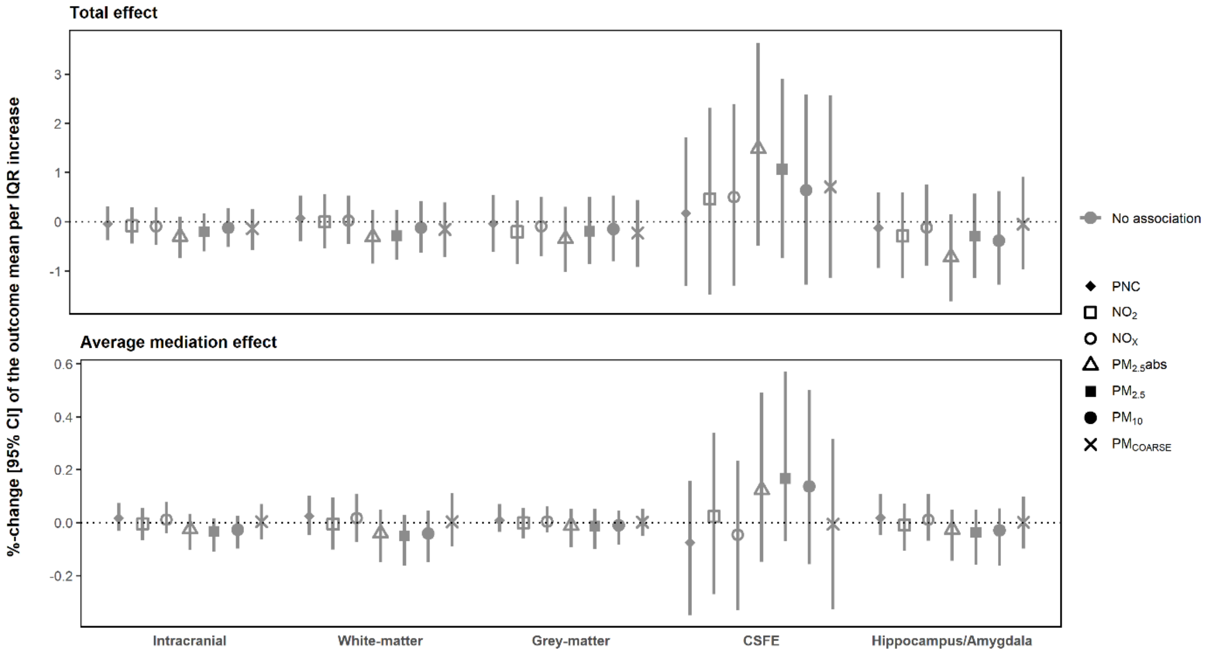

Figure 3: Associations between TRAP and brain volumes, mediated by the mean signal intensity of the olfactory bulb. The upper panel presents %-change and 95%-confidence intervals of the total effect per interquartile-range (IQR) increase in the respective air pollutant. The lower panel presents %-change and 95%-confidence intervals of the average mediation effect per IQR increase in the respective air pollutant. CSFE: cerebro-spinal fluid. Strength of association derived from the mediation analysis: significant: p-value < 0.05. Trend: 0.1 > p-value ≥ 0.05. No association: p-value ≥ 0.1. Models were adjusted for age, sex, BMI, alcohol consumption, smoking, physical activity, marital status and education years. N = 322.

Supplementary Figure 4 – Association between TRAP and brain volumes, mediated by the maximum signal intensity of the olfactory bulb

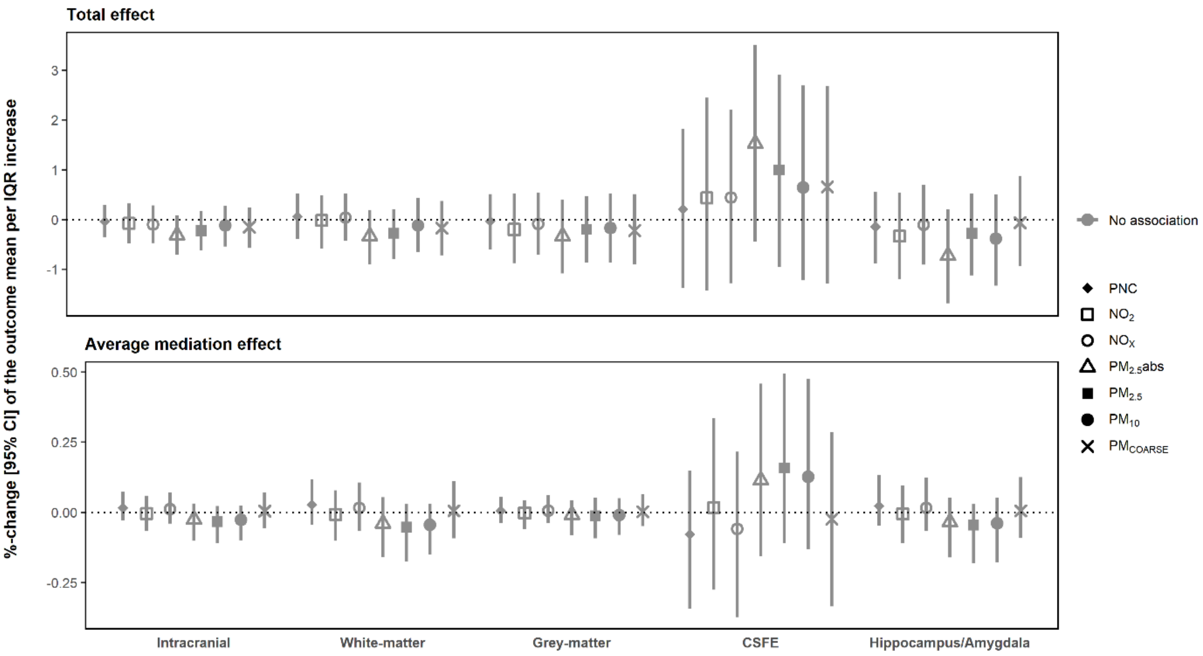

Figure 4: Associations between TRAP and brain volumes, mediated by the maximum signal intensity of the olfactory bulb. The upper panel presents %-change and 95%-confidence intervals of the total effect per interquartile-range (IQR) increase in the respective air pollutant. The lower panel presents %-change and 95%-confidence intervals of the average mediation effect per IQR increase in the respective air pollutant. CSFE: cerebro-spinal fluid. Strength of association derived from the mediation analysis: significant: p-value < 0.05. Trend: 0.1 > p-value ≥ 0.05. No association: p-value ≥ 0.1. Models were adjusted for age, sex, BMI, alcohol consumption, smoking, physical activity, marital status and education years. N = 322.

Supplementary Figure 5 – Association between TRAP and brain volumes, stratified by sex

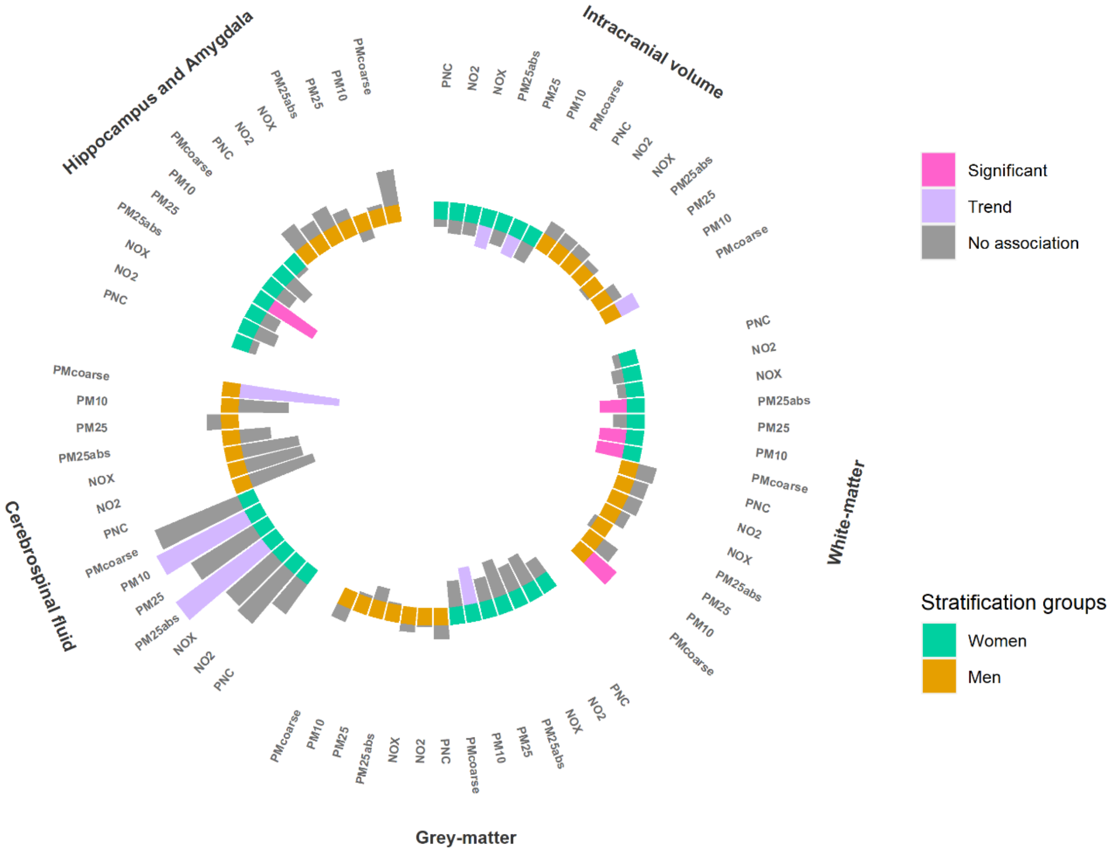

Figure 5: Associations between TRAP and brain volumes per IQR increase, stratified by sex. The height of the bars indicates the effect size (%-change) per interquartile range (IQR) increase in the respective air pollutant. The direction of the bars indicates the direction of the association. Bars toward the center indicate a negative association, bars toward outside a positive association. Strength of association derived from the regression analysis: significant: p-value < 0.05. Trend: 0.1 > p-value ≥ 0.05. No association: p-value ≥ 0.1. Models were adjusted for age, BMI, alcohol consumption, smoking, physical activity, marital status and education years. N = 352.

Supplementary Figure 6 – Association between TRAP and white-matter lesions and cerebral microbleeds, stratified by diabetes status

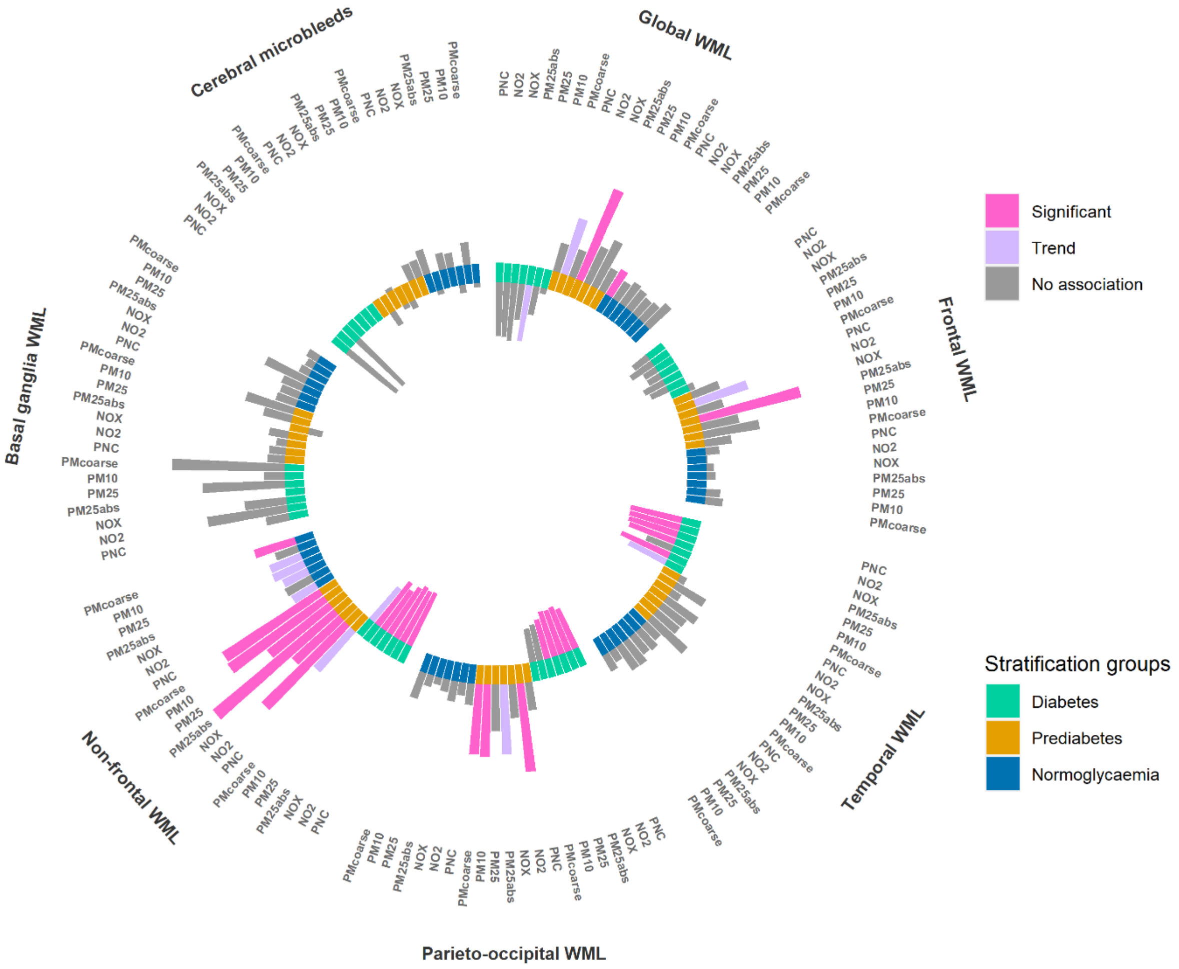

Figure 6: Associations between TRAP and global and region-specific white-matter lesions (WML), and cerebral microbleeds per IQR increase, stratified by diabetes. The height of the bars indicates the odds ratio (OR) per interquartile range (IQR) increase in the respective air pollutant. The direction of the bars indicates the direction of the association. Bars toward the center indicate a negative association (OR <1), bars toward outside a positive association (OR >1). Strength of association derived from the regression analysis: significant: p-value < 0.05. Trend: 0.1 > p-value ≥ 0.05. No association: p-value ≥ 0.1. Models were adjusted for age, BMI, alcohol consumption, smoking, physical activity, marital status and education years. N = 379.

Supplementary Figure 7 – Association between TRAP and white-matter lesions and cerebral microbleeds, stratified by BMI

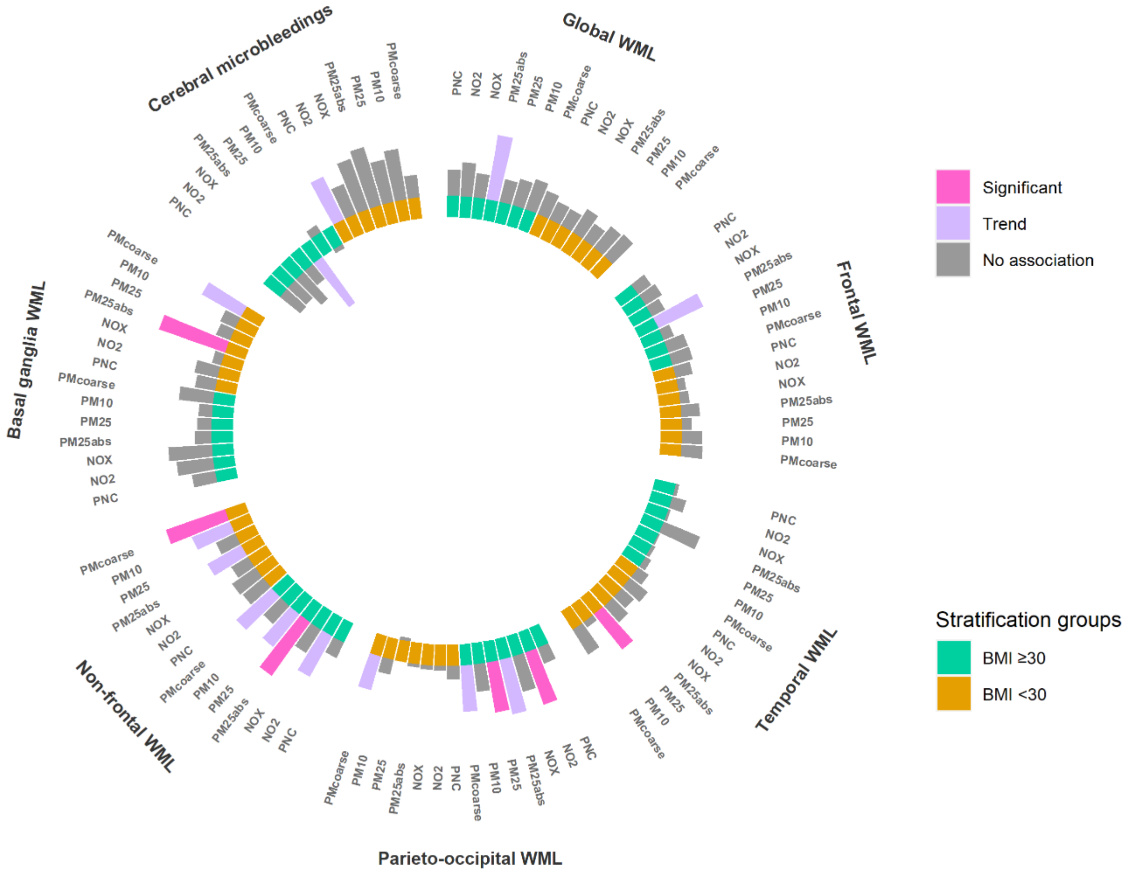

Figure 7: Associations between TRAP and global and region-specific white-matter lesions (WML), and cerebral microbleeds per IQR increase, stratified by BMI. The height of the bars indicates the odds ratio (OR) per interquartile range (IQR) increase in the respective air pollutant. The direction of the bars indicates the direction of the association. Bars toward the center indicate a negative association (OR <1), bars toward outside a positive association (OR >1). Strength of association derived from the regression analysis: significant: p-value < 0.05. Trend: 0.1 > p-value ≥ 0.05. No association: p-value ≥ 0.1. Models were adjusted for age, BMI, alcohol consumption, smoking, physical activity, marital status and education years. N = 379.

Supplementary Figure 8 – Association between TRAP and white-matter lesions and cerebral microbleeds, stratified by hypertension

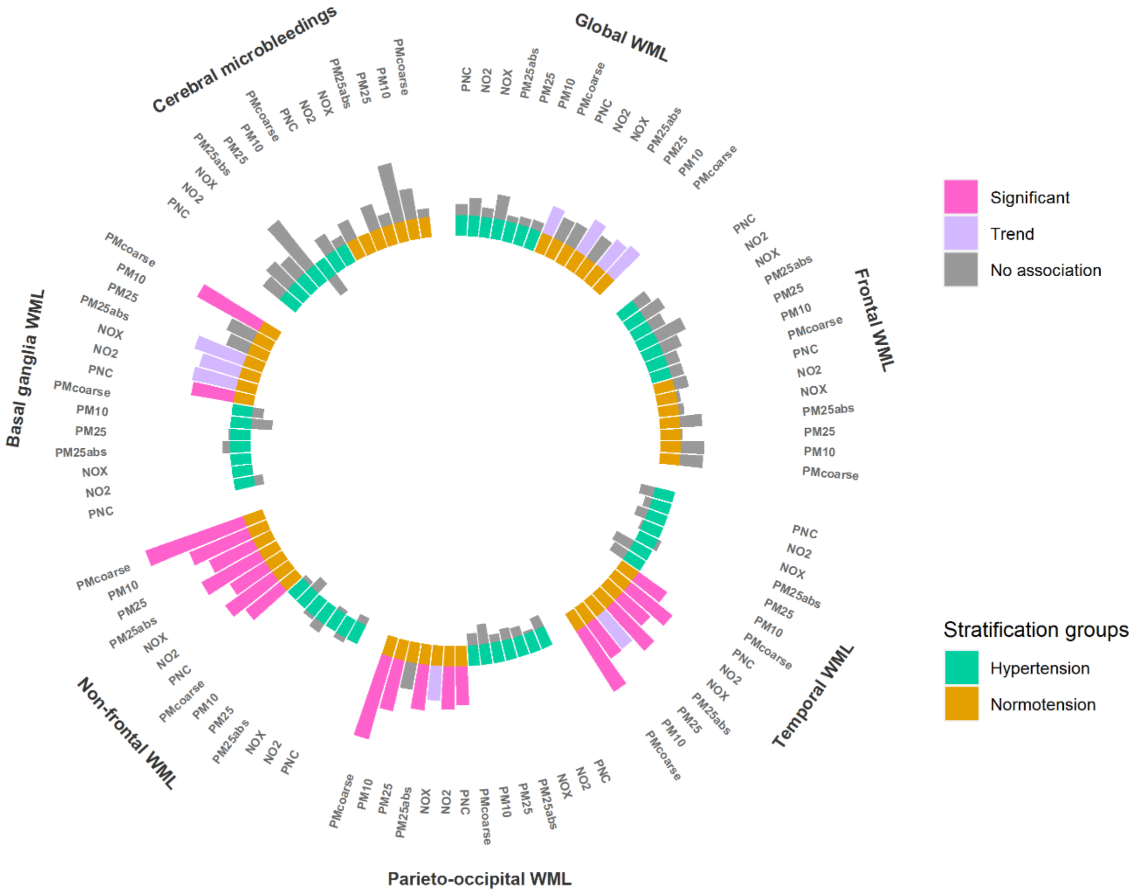

Figure 8: Associations between TRAP and global and region-specific white-matter lesions (WML), and cerebral microbleeds per IQR increase, stratified by hypertension. The height of the bars indicates the odds ratio (OR) per interquartile range (IQR) increase in the respective air pollutant. The direction of the bars indicates the direction of the association. Bars toward the center indicate a negative association (OR <1), bars toward outside a positive association (OR >1). Strength of association derived from the regression analysis: significant: p-value < 0.05. Trend: 0.1 > p-value ≥ 0.05. No association: p-value ≥ 0.1. Models were adjusted for age, BMI, alcohol consumption, smoking, physical activity, marital status and education years. N = 379.

Supplemental material reference list

(1) Galiè, F., Rospleszcz, S., Keeser, D, et al. (2020). Machine-learning based exploration of determinants of gray matter volume in the KORA-MRI study. *Scientific reports*, *10*(1), 8363. https://doi.org/10.1038/s41598-020-65040-x

(2) Beller, E., Keeser, D., Wehn, A., et al. (2019). T1-MPRAGE and T2-FLAIR segmentation of cortical and subcortical brain regions-an MRI evaluation study. *Neuroradiology*, *61*(2), 129–136. https://doi.org/10.1007/s00234-018-2121-2

(3) Xiong, Y., Mok, V., Wong, A., et al. (2010). The age-related white matter changes scale correlates with cognitive impairment. *European journal of neurology*, *17*(12), 1451–1456. https://doi.org/10.1111/j.1468-1331.2010.03078.x

(4) Wahlund, L. O., F. Barkhof, F. Fazekas, L., et al. (2001). 'A new rating scale for age-related white matter changes applicable to MRI and CT', Stroke, 32: 1318-22.

(5) Bamberg, F., Hetterich, H., Rospleszcz, S., et al.. (2017). Subclinical Disease Burden as Assessed by Whole-Body MRI in Subjects With Prediabetes, Subjects With Diabetes, and Normal Control Subjects From the General Population: The KORA-MRI Study. *Diabetes*, *66*(1), 158–169. https://doi.org/10.2337/db16-0630

(6) Grosu, S., Rospleszcz, S., Hartmann, F., et al.. (2021). Associated factors of white matter hyperintensity volume: a machine-learning approach. *Scientific reports*, *11*(1), 2325. https://doi.org/10.1038/s41598-021-81883-4
